# Tribbles Pseudokinase 3 Contributes to Cancer Stemness of Endometrial Cancer Cells by Regulating β-Catenin Expression

**DOI:** 10.3390/cancers12123785

**Published:** 2020-12-15

**Authors:** Wen-Ling Wang, Guan-Ci Hong, Peng-Ju Chien, Yu-Hao Huang, Hsueh-Te Lee, Po-Hui Wang, Yueh-Chun Lee, Wen-Wei Chang

**Affiliations:** 1 School of Biomedical Sciences, Chung Shan Medical University, Taichung 40201, Taiwan; u8966003.lin@gmail.com (W.-L.W.); cream8515@gmail.com (G.-C.H.); chienpengju@gmail.com (P.-J.C.); bms0217062@gmail.com (Y.-H.H.); 2 Institute of Anatomy and Cell Biology, School of Medicine, National Yang Ming University, Taipei 11221, Taiwan; incubator.lee@ym.edu.tw; 3 Institute of Medicine, Chung Shan Medical University, Taichung 40201, Taiwan; wang082160@gmail.com; 4 Department of Obstetrics and Gynecology, Chung Shan Medical University Hospital, Taichung 40201, Taiwan; 5 School of Medicine, Chung Shan Medical University, Taichung 40201, Taiwan; 6 Radiation Oncology Department, Chung Shan Medical University Hospital, Taichung 40201, Taiwan; 7 Department of Medical Research, Chung Shan Medical University Hospital, Taichung 40201, Taiwan

**Keywords:** tribbles pseudokinase 3, endometrial cancer, cancer stem cells, β-catenin, epithelial–mesenchymal transition

## Abstract

**Simple Summary:**

Endometrial cancer (EC) is the second most common female malignancy worldwide, but the pathogenesis is not fully understood. Tribbles pseudokinase 3 (TRIB3) is a kind of scaffold protein that may regulate multiple cellular processes by organizing binding partner proteins involving signaling transduction pathways. The goal of this study is to investigate if TRIB3 is involved in the malignant features of EC. Our data demonstrate that TRIB3 positively regulates the cancer stem-cell activity and in vivo tumorigenicity of EC cells by modulating β-catenin signaling through directly interacting with the ELF4 transcription factor. Our results could lead to new insight for developing a novel therapeutic strategy for EC by targeting TRIB3.

**Abstract:**

Endometrial cancer (EC) is the second most common gynecological malignancy worldwide. Tribbles pseudokinase 3 (TRIB3) is a scaffolding protein that regulates intracellular signal transduction, and its role in tumor development is controversial. Here, we investigated the biological function of TRIB3 in EC. We found that the messenger RNA (mRNA) expression level of TRIB3 was significantly and positively correlated with shorter overall survival of EC patients in The Cancer Genome Atlas database. The protein expression of TRIB3 was found to be significantly increased in EC cancer stem cells (CSCs) enriched by tumorsphere cultivation. Knockdown of TRIB3 in EC cells suppressed tumorsphere formation, the expression of cancer stemness genes, and the in vivo tumorigenesis. The expression of β-catenin at both the protein and the mRNA levels was downregulated upon TRIB3 silencing. TRIB3 was found to interact with E74 Like ETS transcription factor 4 (ELF4) in the nucleus and bound to ELF4 consensus sites within the catenin beta 1 (*CTNNB1*) promoter in EC cell lines. These data indicated that TRIB3 may regulate *CTNNB1* transcription by enhancing the recruitment of ELF4 to the *CTNNB1* promoter. In conclusion, our results suggest that TRIB3 plays an oncogenic role in EC and positively regulates the self-renewal and tumorigenicity of EC-CSCs. Targeting TRIB3 is considered as a potential therapeutic strategy in future EC therapy.

## 1. Introduction

Endometrial carcinomas (ECs) are cancers that develop from the cells of the inner lining of the uterus (the endometrium), and they are the second most common gynecological malignancy and the 15th most common cancer worldwide [1]. The total number of ECs in Taiwan increased by 5.7-fold from 1991 to 2010 [2], and they were ranked sixth among female cancers in 2016. ECs are divided into two histologic types. Type I ECs are mostly endometrioid adenocarcinomas that are estrogen-dependent, and they account for 75–80% of ECs. Type II ECs are characterized by nonendometrioid histology with no expression of hormone receptors, and they have poor prognoses [3]. The risk factors for ECs include adiponectin, estrone, interleukin-1 receptor antagonist, tumor necrosis factor-alpha, and triglycerides, as determined by a study from Europe [4]. Although the overall survival of endometrial cancer is good, the prognosis of recurrent disease is poor, and the recurrence rate was reported to be significantly increased in later FIGO (International Federation of Gynecology and Obstetrics) stages [5]. In addition, catenin beta 1 (*CTNNB1*) mutations were observed in 20–25% of ECs [6] and shorter overall survival was observed in low-grade ECs harboring the activation of wingless-type MMTV integration site family member (Wnt)/β-catenin target genes, including *MYC* and cyclin D1 (*CCND1*) [7].

Tribbles pseudokinase 3 (TRIB3) is a pseudokinase protein and is classified as a scaffolding protein that participates in cellular signaling pathways [8]. Through its pseudokinase domain, TRIB3 has been reported to interact with serine/threonine kinase Akt (AKT1), CCAAT enhancer binding protein alpha (C/EBPα), and mitogen-activated protein kinase kinase (MAPKK) [9]. TRIB3 has been demonstrated to reduce adipogenesis [10] and insulin signaling [11], but its role in cancer development is controversial. A tumor suppressor function of TRIB3 has been proposed via the inactivation of AKT1 or nuclear factor kappa B subunit (NF-κB) [12] through direct binding. However, TRIB3 has been reported to be overexpressed in several cancer types, such as breast cancer [13,14] and colorectal cancer [15,16]. TRIB3 has been shown to interact with SMAD family member 3 (SMAD3) to promote transforming growth factor beta (TGFβ)-induced tumor cell migration and invasion [17]. TRIB3 has also been demonstrated to interact with p62, leading to the inhibition of autophagic degradation of several oncogenic proteins. Disruption of the interaction between TRIB3 and p62 resulted in tumor suppression in an animal model [18]. In acute promyelocytic leukemia, TRIB3 has been demonstrated to promote malignancy through stabilization of the promyelocytic leukemia-retinoic acid receptor alpha (PML-RARα) oncoprotein [19]. Recently, TRIB3 was reported to regulate the β-catenin pathway in colorectal cancer [15] and to suppress forkhead box protein O1 (FOXO1) phosphorylation in breast cancer [13] by helping to maintain cancer stem cells (CSCs).

CSCs are considered the basis of tumor initiation and are the cells responsible for enabling drug resistance and metastasis [20,21]. To date, CSCs have been identified in many solid tumors, including those of the brain, colon, head and neck, liver, lung, ovary, pancreas, and prostate [21,22], and targeting CSCs has been considered the key for successful cancer treatment [21,23]. In ECs, the markers for CSCs include aldehyde dehydrogenase (ALDH), CD44, CD55, CD117, and CD133 [24]. The high expression of ALDH1A1 was reported to be associated with poor survival in EC patients and the tumorsphere derived from clinical EC specimens displayed high ALDH activity [25]. In addition to CSC markers, targeting cancer stemness genes has been found to have potential for EC treatment, such as B-lymphoma Mo-MLV insertion region 1 (BMI1) [26] or homeobox transcription factor Nanog (NANOG) [27]. In the present study, we first discovered that the expression of TRIB3 messenger RNA (mRNA) was upregulated in EC tissues as the stage increased, and its expression was positively correlated with shorter survival time in EC patients in The Cancer Genome Atlas (TCGA) database. TRIB3 expression was increased in tumorspheres of AN3CA or HEC1A EC cell lines, and the knockdown of TRIB3 in AN3CA or HEC1A EC cell lines caused growth inhibition. The inhibition of tumorsphere formation capability, epithelial–mesenchymal transition (EMT), cancer stemness genes, and tumorigenicity was observed in EC cells when TRIB3 was knocked down. We further discovered that the knockdown of TRIB3 downregulated β-catenin activity through both transcriptional and translational mechanisms. The regulation of the β-catenin pathway by TRIB3 was also observed in primary EC cells derived from Taiwanese patients.

## 2. Results

### 2.1. The TRIB3 Expression Level Is Positively Correlated with Shorter Overall Survival in EC Patients

**Figure 1:**
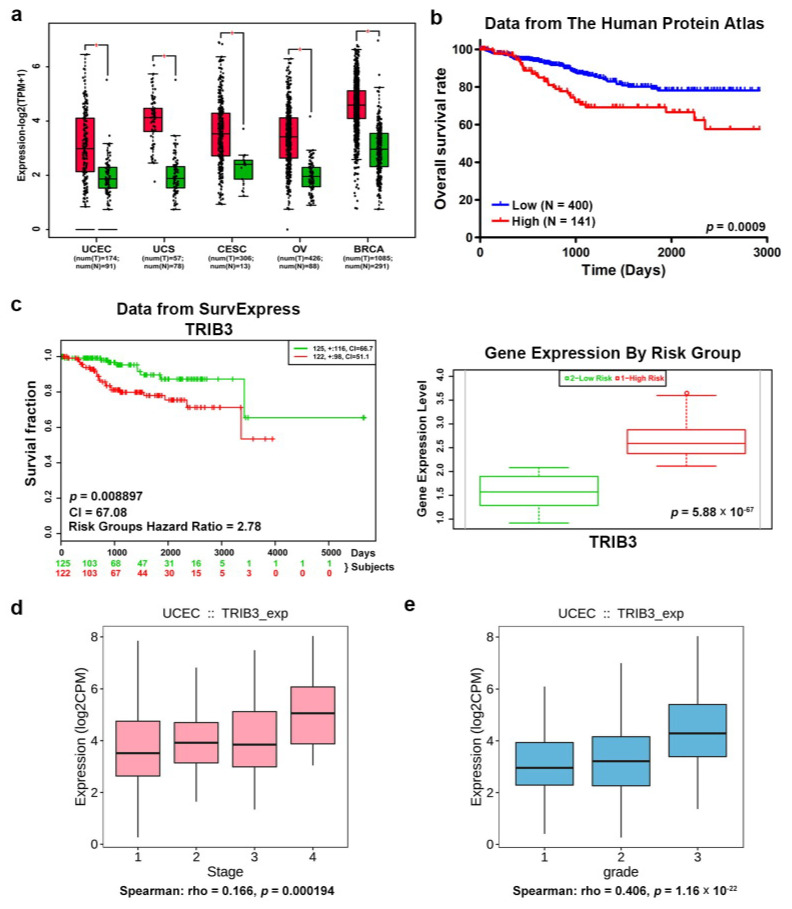
Tribbles pseudokinase 3 (TRIB3) expression is positively associated with shorter overall survival of endometrial cancer (EC) patients. (**a**) The TRIB3 expression levels among cancerous (red color) and normal (green color) female tissues in The Cancer Genome Atlas (TCGA) database including uterine corpus endometrial carcinoma (UCEC), uterine carcinosarcoma (UCS), cervical squamous cell carcinoma and endocervical adenocarcinoma (CESC), ovarian serous cystadenocarcinoma (OV), and breast invasive carcinoma (BRCA) were analyzed and plotted using the Gene Expression Profiling Interactive Analysis (GEPIA) website. The cutoff *p*-value was set to 0.01. (**b**) The correlation between TRIB3 expression and overall survival of UCEC patients in TCGA database was analyzed using the Kaplan–Meier plotter from the Human Protein Atlas. (**c**) The correlation between TRIB3 and overall survival of UCEC patients was also analyzed using the SurvExpress website. The red box and green box in the right panel represent the high-risk group and low-risk group, respectively. (**d**,**e**) The correlations of TRIB3 expression with clinical stages (**d**) and histology grades (**e**) of the UCEC dataset in TCGA database were analyzed using the TISDB website.

Using Gene Expression Profiling Interactive Analysis (GEPIA) to analyze the RNA Seq data from TCGA [28], we first found that, compared to normal tissue, TRIB3 expression was upregulated in tumor tissues of cancers in women, including breast (BRCA), cervical (CESC), ovarian (OV), uterine carcinosarcoma (UCS), and uterine corpus endometrial carcinoma (UCEC) (Figure 1a). We next analyzed the correlation between TRIB3 and patient survival from the Human Protein Atlas [29] EC patients with high levels of TRIB3 expression displayed shorter overall survival (Figure 1b, *p* = 0.0009). Similar results were found in SurvExpress [30] (Figure 1c, *p* = 0.008897, concordance index = 67.08, risk groups hazard ratio = 2.78). In addition, TRIB3 expression was significantly higher in the high risk group of UCEC samples (Figure 1c, *p* = 5.88 × 10^−67^) analyzed from the SurvExpress website and increased with increasing cancer stage (Figure 1d, *R* = 0.166, *p* = 0.00194) and histology grade (Figure 1e, *R* = 0.406, *p* = 1.16 × 10^−22^) according to the analysis results from the TISIDB website [31]. These results demonstrated that the mRNA expression level of TRIB3 is positively correlated with a shorter overall survival time among EC patients and suggest that TRIB3 is a potential prognostic factor in EC and that it may contribute to its progression.

### 2.2. TRIB3 Knockdown Suppresses the Growth of EC Cells

**Figure 2:**
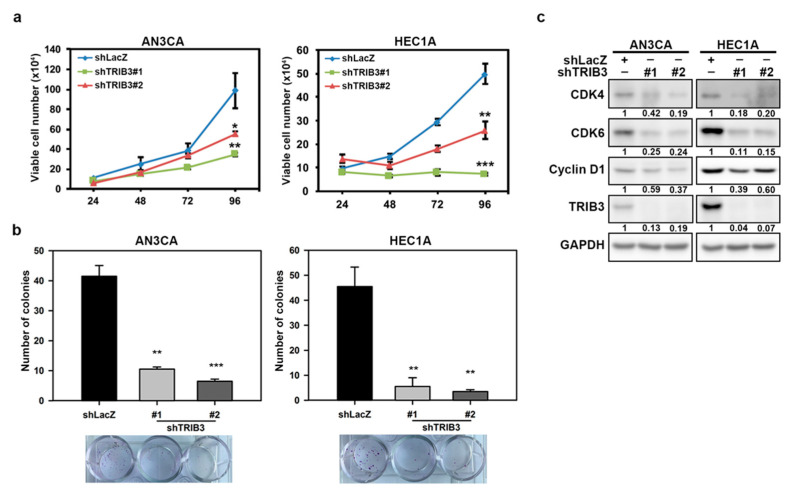
TRIB3 silencing decreased cell growth through the disruption of cyclin D1 and cyclin-dependent kinase (CDK) 4/6 expression. AN3CA or HEC1A cells were transduced with shLacZ or TRIB3-specific short hairpin RNAs (shRNAs) carrying lentiviruses and selected with 2 μg/mL puromycin for 3 days. (**a**) The survived cells were then seeded into a 12-well plate at 1 × 10^4^ cells/well and the cell number was counted with a trypan blue exclusion assay every 24 h. (**b**) The cells were seeded into a 12-well plate at 250 cells/well and incubated for 7 days. The cell colonies were visualized and counted after crystal violet staining. (**c**) Specific amounts (25 μg) of total cellular proteins from AN3CA (**c left panel**) or HEC1A (**c right panel**) cells transduced with shRNA carrying lentiviruses were subjected to determine indicated proteins by Western blot analysis. * *p* < 0.05; ** *p* < 0.01; *** *p* < 0.001.

To explore the biological functions of TRIB3 in EC cells, we first employed a loss-of-function approach to knock down TRIB3 by lentiviral delivery of specific short hairpin RNAs (shRNAs); subsequently, a cell counting assay and a clonogenic assay were used to determine the effect of TRIB3 knockdown on cell proliferation. The growth analysis of AN3CA or HEC1A cells after knockdown of TRIB3 was performed using a trypan blue exclusion assay, and the results revealed that a significantly decreased cell number at 96 h was observed in both AN3CA (*p* = 0.00344 for shTRIB3#1 and *p* = 0.0137 for shTRIB3#2) and HEC1A (*p* = 6.17 × 10^−5^ for shTRIB3#1 and *p* = 0.0017 for shTRIB3#2) cells when compared with the control shLacZ lentivirus transduced cells (Figure 2a). Consistently, the results of the clonogenic assay revealed that both AN3CA and HEC1A cell lines with TRIB3 knockdown resulted in a significant reduction in colony number (Figure 2b). These results indicated that TRIB3 plays an important role in mediating the proliferation of EC cells. We next detected the expression of cyclins and cyclin-dependent kinases (CDKs) by Western blot. In both TRIB3-silenced AN3CA and HEC1A cells, the expression of cyclin D1, CDK4, and CDK6 was notably decreased (Figure 2c). Taken together, the knockdown of TRIB3 in EC cells disturbed the activation of cell-cycle checkpoint molecules and led to the inhibition of cell growth.

### 2.3. Silencing of TRIB3 Attenuates EC Cell Migration and Invasion and Decreases the Expression Levels of EMT-Associated Factors

**Figure 3:**
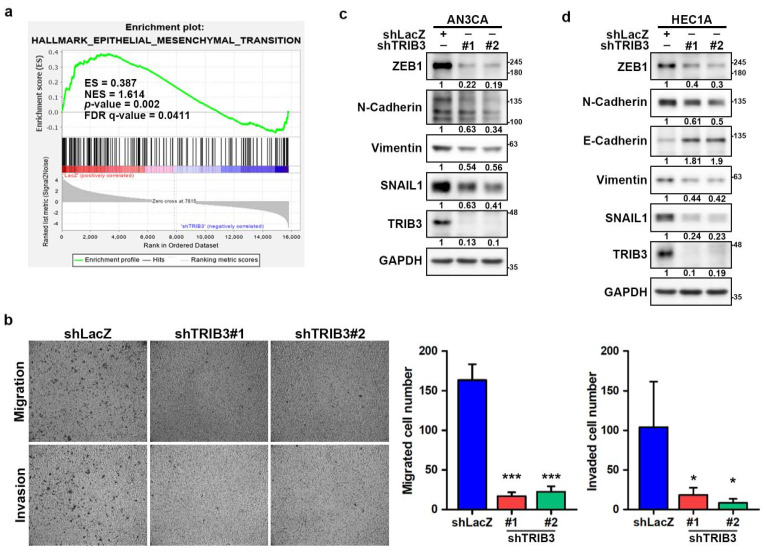
TRIB3 knockdown inhibited cell migration/invasion and suppressed the epithelial–mesenchymal transition (EMT)-related molecules of EC cells. (**a**) Gene set enrichment analysis (GSEA) of RNA-seq data identified an enrichment of EMT genes signatures in shLacZ control (red) vs. TRIB3-knockdown (blue) HEC1A cells. (**b**) AN3CA cells transduced with shLacZ or TRIB3-specific shRNAs carrying lentiviruses and selected with 2 μg/mL puromycin for 3 days. Cell migration or invasion abilities were determined by transwell assay through seeding of 1 × 10^5^ in the upper chamber. After incubation for 24 h, migrated and invaded cells were visualized (**left panel**) and counted (**right panel**) after staining with 0.1% crystal violet. Data are presented as the mean ± SD from three files of wells. * *p* < 0.05, and *** *p* < 0.001 when compared to shLacZ control. (**c**,**d**) AN3CA (**c**) or HEC1A (**d**) cells were transduced with shLacZ or TRIB3-specific shRNAs (#1 or #2) carrying lentiviruses and selected with 2 μg/mL puromycin for 3 days. Specific amounts (25 μg) of total cellular proteins were used for determination of indicated proteins by Western blot analysis.

Hua et al. previously reported that TRIB3 could be involved in tumor invasion and migration [17]. To verify the association of TRIB3 expression with the EMT process in EC cells, RNA-seq analysis of TRIB3 knockdown HEC1A cells was performed, and gene set enrichment analysis (GSEA) revealed that TRIB3 was positively correlated with EMT signaling (NES = 1.614, FDR *q*-value = 0.0411) (Figure 3a). We next performed a transwell assay to assess the effect of TRIB3 on the migratory and invasive abilities of EC cells and found that the knockdown of TRIB3 inhibited cell migration and invasion of AN3CA cells (Figure 3b). EMT is an important process in the conversion of early-stage tumors into invasive malignancies, and it is regulated by a panel of transcription repressors that suppress the expression of epithelial markers such as E-cadherin [32]. We next examined the effect of TRIB3 on the expression of EMT markers and found that, compared to the levels observed in control shLacZ transduced cells, the expression of N-cadherin, zinc finger E-box binding homebox 1 (ZEB1), Vimentin, and sanil family transcriptional repressor 1 (SNAI1) was markedly decreased in TRIB3-silenced AN3CA (Figure 3c) and HEC1A (Figure 3d) cells. The expression of E-cadherin was notably increased in TRIB3-silenced HEC1A cells (Figure 3d). These results indicated that TRIB3 expression may contribute to the mesenchymal phenotypes of EC cells to promote metastatic potential.

### 2.4. Knockdown of TRIB3 Decreases the Self-Renewal Capability and Tumorigenicity of EC-CSCs

**Figure 4:**
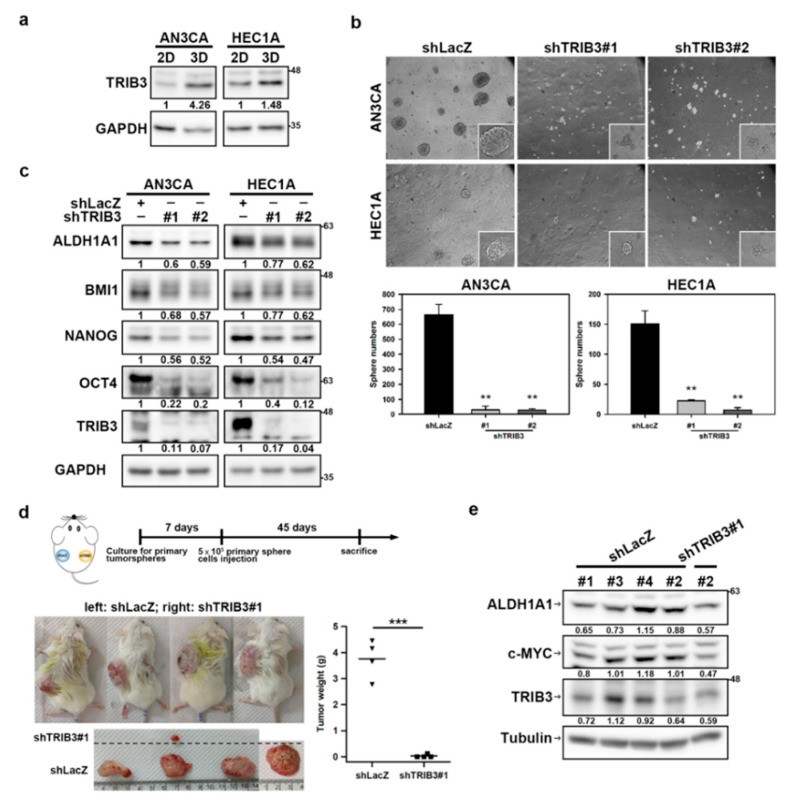
Knockdown of TRIB3 impaired self-renewal capability and tumorigenicity of EC cancer stem cells (CSCs). (**a**) Protein expression levels of TRIB3 in conventional adherence (two-dimensional, 2D) or tumorsphere (3D) cultured AN3CA or HEC1A cells were determined by Western blot. (**b**,**c**) AN3CA or HEC1A cells were transduced with shLacZ or TRIB3 specific shRNA (#1 or #2) carrying lentiviruses and selected with 2 μg/mL puromycin for 3 days. The survived cells were then performed tumorsphere cultivation for 7 days followed by picturing (upper panel) and counting the number (lower panel) of formed tumorspheres with inverted light microscopy. Data represent the mean ± SD. ** *p* < 0.01, *** *p* < 0.001 when compared to shLacZ transduced cells (**b**). Specific amounts (25 μg) of total cellular proteins from shRNA transduced AN3CA or HEC1A cells were subjected to determine the expression of indicated proteins by Western blot analysis (**c**). (**d**) NOD/SCID mice were injected subcutaneously with 5 × 10^5^ cells of AN3CA-shLacZ or AN3CA-shTRIB3 primary tumorsphere cells and the tumor formation was monitored until day 45 post injection (**upper panel**). The central panel shows the appearance of tumor-bearing NOD/SCID mice implanted with AN3CA-shLacZ (**left site**) or AN3CA-shTRIB3 cells (**right site**). The lower panel indicates the excised tumor masses followed by weighing. *** *p* < 0.001 compared to control shLacZ-transfected group. (**e**) The expressions of aldheyde dehydrogenase (ALDH1A1), c-MYC, and TRIB3 protein in xenografted tumors from one experimental repeat with a small engrafted tumor after injection of TRIB3-knockdown AN3CA cells were examined by Western blotting analysis.

EC-CSCs have been reported to drive metastasis, chemotherapy resistance, and disease relapse [24], and EC-CSCs could be enriched with a three-dimensional (3D) tumorsphere cultivation method. With tumorsphere cultivation, we found that TRIB3 expression was increased in both AN3CA and HEC1A tumorspheres compared to those from conventional 2D adhered culture with serum-containing medium (Figure 4a). Knockdown of TRIB3 in AN3CA or HEC1A cells obviously reduced the size and number of tumorspheres (Figure 4b). The expression of ALDH1A1, a CSC marker, and the cancer stemness factors including BMI1, NANOG, and octamer-binding transcription factor (OCT4) were also reduced in both AN3CA and HEC1A cells after TRIB3 silencing (Figure 4c). We further examined the importance of TRIB3 expression in the tumorigenicity of EC-CSCs. A single-cell suspension of primary tumorspheres derived from AN3CA cells was collected for transduction with shLacZ- or sh-TRIB3#1-carrying lentiviruses, and the cells were subcutaneously injected into the interscapular area of NOD/SCID mice; tumor growth was monitored thereafter (Figure 4d). The incidence rate of tumor formation was 12.5% (one out of eight mice obtained from two independent experiments) for sh-TRIB3-transduced tumorsphere cells and 100% for the shLacZ group (Figure 4d). Furthermore, the tumor weight of TRIB3 knockdown EC tumorsphere cells was significantly reduced (Figure 4d). We further checked the protein expression of TRIB3, ALDH1A1, and c-MYC in lysates of xenografted tumors and results showed that TRIB3 was slightly decreased in the only one small engrafted tumor of TRIB3-knockdown AN3CA cells, while the expression levels of ALDH1A1 and c-MYC were greatly reduced (Figure 4e). Taken together, these results suggest that TRIB3 contributes to the self-renewal capability and tumorigenicity of EC-CSCs.

### 2.5. TRIB3 Participates in the Expression and Activity of β-Catenin in EC Cells

**Figure 5:**
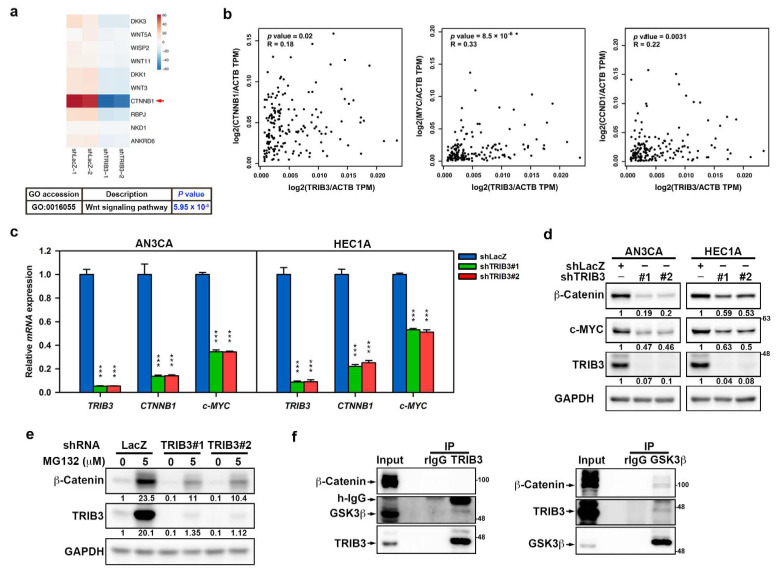
β-catenin expression in EC cells was regulated by TRIB3 in transcriptional and translational mechanisms. (**a**) HEC1A cells were transduced with shLacZ and TRIB3-specific shRNA (#1) carrying lentiviruses and selected with 2 μg/mL puromycin for 3 days followed by RNA-seq analysis in duplicate. The upper panel shows the genes downregulated in HEC1A cells with TRIB3 knockdown significantly enriched in the WNT signaling pathway. Heat map of the WNT/β-catenin signal-associated genes altered in TRIB3-knockdown HEC1A cells compared to shLacZ group. *CTNNB1* is highlighted with a red arrow (lower panel). (**b**) Pearson’s correlation analysis of TRIB3 and *CTNNB1*(left panel), as well as β-catenin downstream genes, *c-MYC* (middle panel) and *CCND1* (right panel), in UCEC patients were analyzed and plotted using the GEPIA website (http://gepia.cancer-pku.cn/index.html). (**c**,**d**) AN3CA (left panel) or HEC1A (right panel) cells were transduced with lentiviruses carrying with shLacZ (blue) or TRIB3-specific shRNAs (#1, green; #2, red) for 3 days. The relative mRNA levels of *TRIB3*, *c-MYC*, and *CTNNB1* were determined by SYBR-Green-based qRT-PCR (**c**). Data are presented as fold changes (mean ± SD) relative to shLacZ from three individual experiments. *** *p* < 0.001 when compared to shLacZ. (**d**) Specific amounts (25) μg of total cellular proteins were used for determination of β-catenin, c-Myc, or TRIB3 expression by Western blot analysis. (**e**) shRNA transduced AN3CA cells were treated with 5 μM of MG-132 for 24 h, and 25 μg of total cellular proteins were harvested for analyzing the expression of TRIB3 and β-catenin by Western blot. (**f**) Specific amounts (0.5 mg) of total cellular proteins from AN3CA cells were used for immunoprecipitation with an anti-TRIB3 antibody or control IgG following by the detection of β-catenin and GSK3β in pull-down proteins by Western blot (left panel). Whole-cell extracts from AN3CA cells were immunoprecipitated with anti-GSK3b antibody or rabbit IgG and blotted with anti-β-catenin and anti-TRIB3 antibodies (right panel).

The WNT/β-catenin signaling pathway plays an important role in EMT and in the self-renewal properties of EC-CSCs [24]. Treatment of AN3CA and HEC1A cells with the β-catenin inhibitors CCT-031374 or PNU-74654 resulted in a dose-dependent decrease in cell viability (Figure S1a,b). To determine whether TRIB3 promoted EC tumorigenesis through the WNT/β-catenin pathway, we analyzed the gene expression profiles from RNA sequencing. Through Gene Ontology (GO) analysis, the WNT signaling pathway was found to be suppressed in TRIB3-knockdown HEC1A cells compared to shLacZ-transduced control cells (*p* = 0.00000595) (Figure 5a, upper panel). Genes involved in the Wnt signaling pathway, including *CTNNB1*, ankyrin repeat domain-containing protein 6 (*ANKRD6*), *WNT3*, *WNT5A, WNT11*, WNT1-inducible-signaling pathway protein 2 (*WISP2*), dickkopf-related protein 1 (*DKK1*), *DKK3*, recombining binding protein suppressor of hairless (*RBPJ*), and protein naked cuticle homolog 1(*NKD1*)*,* were downregulated in TRIB3-knockdown HEC1A cells compared to shLacZ cells (Figure 5a, lower panel). We found that *CTNNB1* was the most abundant among these genes and that it was strongly repressed in TRIB3-knockdown cells. We next used the GEPIA data base to investigate the relationship of the mRNA expression between β-catenin, encoded by *CTNNB1* gene, and *TRIB3* among UCEC tumors. The result revealed that there was a positive correlation between *TRIB3* and *CTNNB1* mRNA expression among UCEC tumors (Figure 5b left panel). Furthermore, *MYC* and *CCND1*, which are the well-known downstream molecules of the β-catenin pathway, had a positive correlation with *TRIB3* in the same UCEC dataset (Figure 5b, middle panel and right panel). The data suggest that TRIB3 may regulate β-catenin to participate in the WNT signaling pathway in ECs. Thus, we decided to examine whether TRIB3 participated in the regulation of β-catenin expression and found that the knockdown of TRIB3 in both AN3CA and HEC1A cells resulted in a significant reduction in *CTNNB1* mRNA (Figure 5c) and β-catenin protein (Figure 5d). The expression of c-MYC was decreased in both AN3CA and HEC1A cells after TRIB3 silencing at the mRNA (Figure 5c) and protein (Figure 5d) levels, which indicated that β-catenin activity was suppressed after TRIB3 knockdown. The regulation of cellular β-catenin is mainly regulated by the proteasomal pathway [33]. Treatment with MG-132, an inhibitor of the proteasome, at a noncytotoxic dosage slightly prevented the reduction in β-catenin expression caused by TRIB3 knockdown in AN3CA cells (Figure 5e). We next examined whether TRIB3 could interact with β-catenin or GSK3β. Using immunoprecipitation analysis, we show that β-catenin was not pulled down with an anti-TRIB3 antibody, but the coprecipitation of GSK3β was observed in AN3CA cells (Figure 5f, left panel). The physical interaction between TRIB3 and GSK3β was also verified by immunoprecipitation with an anti-GSK3β antibody (Figure 5f, right panel). These results suggest that TRIB3-mediated β-catenin expression could partially result from GSK3β binding and the subsequent prevention of proteasomal degradation of β-catenin.

### 2.6. TRIB3 Interacts with ELF4 to Transcriptionally Regulate the Expression of β-Catenin

**Figure 6:**
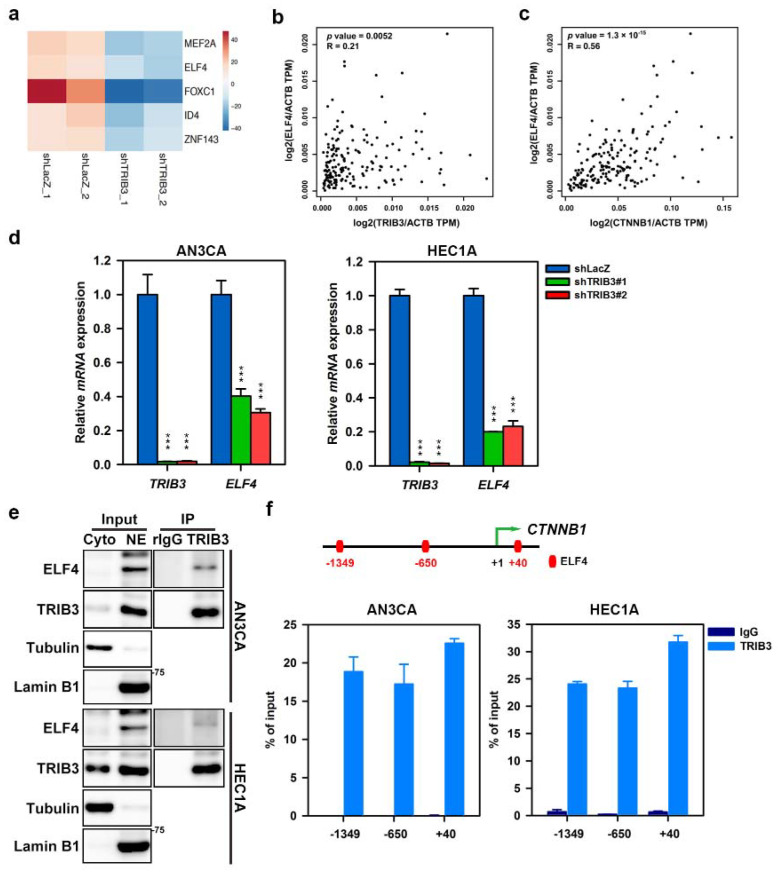
TRIB3 complexes with ELF4 in the nucleus of EC cells and binds to the *CTNNB1* promoter. (**a**) The alterations of putative transcription factors of *CTNNB1* promoter in TRIB3-knockdown HEC1A cells. The heatmap was drawn with the ClustVis web tool using the data from two independent replicates. (**b**,**c**) Pairwise correlations between TRIB3 and ELF4 (**b**) and between ELF4 and *CTNNB1* (**c**) in UCEC dataset of TCGA were analyzed and plotted using the GEPIA website. (**d**) The mRNA expression of *ELF4* in TRIB3-knockdown AN3CA (left panel) and HEC1A (right) cells was analyzed by SYBR-Green-based qRT-PCR. *** *p* < 0.001 when compared to shLacZ group. (**e**) AN3CA and HEC1A cells were pretreated with 2 μM MG132 for 18 h. Then, 200 μg of nuclear extract was used for immunoprecipitation with an anti-TRIB3 antibody or normal rabbit IgG (rIgG) followed by Western blot analysis of ELF4 and TRIB3 expression. Ctyo, cytosolic fraction; NE, nuclear extract. (**f**) The chromatins of AN3CA or HEC1A cells were cross-linked, sonicated into fragments, incubated with 3 μg of anti-TRIB3 antibody (TRIB3) or normal rabbit IgG (IgG), and precipitated with Protein-G-conjugated magnetic beads. The DNA fragments of *CTNNB1* promoter containing the putative ELF4 binding motif (red bars in upper panel) were detected using the qPCR method with three specific primer sets.

We next investigated the transcriptional regulation of β-catenin by TRIB3. After analyzing the *CTNNB1* promoter in the Eukaryotic Promoter Database [34], we found that there were several putative binding sites of ETS transcription factor 4 (ELF4) and myocyte-specific enhancer factor 2A (MEF2A), inhibitor of DNA binding 4 (ID4), forkhead box C1 (FOXC1), and zinc finger protein 143 (ZNF143). The downregulation of these genes was observed in the RNA-seq data of TRIB3-knockdown HEC1A cells (Figure 6a). Through mRNA expression correlation analysis on the GEPIA website, we found that the expression of TRIB3 was positively correlated with ELF4 and MEF2A in UCEC and was statistically significant (Figure 6b and Figure S2a). The expression of *CTNNB1* was also positively correlated with ELF4 and MEF2A in the same samples of patient with UCEC (Figure 6c and Figure S2b). The data suggest that TRIB3 could cooperate with ELF4 and MEF2A to facilitate the expression of *CTNNB1* in EC cells. We further confirmed that the mRNA expression of *ELF4* and *MEF2A* was downregulated in TRIB3-knockdown AN3CA (Figure 6d left panel and Figure S2c,) and HEC1A cells (Figure 6d right panel and Figure S2c). Using a coimmunoprecipitation assay, we demonstrated the physical interaction between TRIB3 and ELF4 in the nucleus in both AN3CA and HEC1A cells (Figure 6e). With the chromatin immunoprecipitation assay, the DNA fragments of *CTNNB1* promoter containing the putative ELF4 binding site (–GGAAG– or –CTTCC–) [35] were amplified using the qPCR method after immunoprecipitating with anti-TRIB3 antibody (Figure 6f). These data suggest that TRIB3 could transcriptionally regulate β-catenin expression by recruiting ELF4 to the *CTNNB1* promoter.

### 2.7. The Regulation Role of TRIB3 in β-Catenin and Cancer Stemness Genes Is Observed in Patient-Derived Primary EC Cells

**Figure 7:**
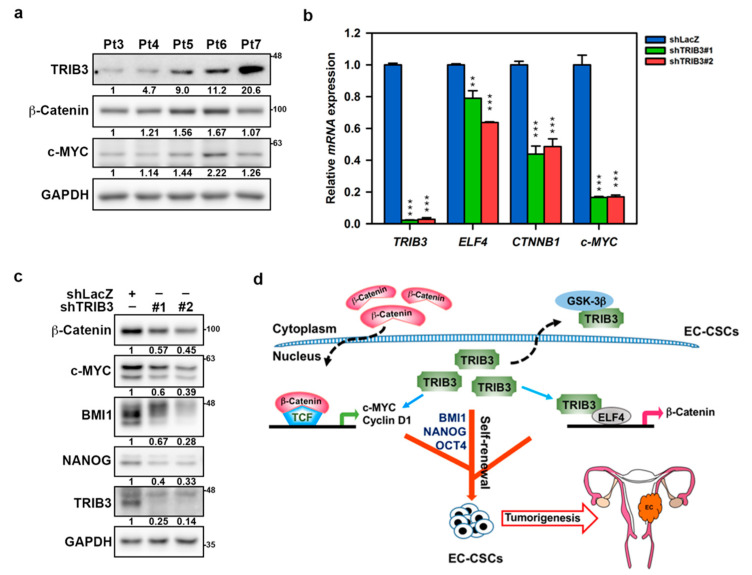
TRIB3 regulates the expression of β-catenin and other cancer stemness genes in patient-derived EC cells. (**a**) Specific amounts (25 μg) of total cellular proteins from primary EC cells derived from five Taiwanese patients were used for examining the expression of TRIB3, c-Myc, and β-catenin by Western blot analysis. (**b**,**c**) EMC5 cells (established primary EC cells from patient No. 5 with clinical grade 3) were established and transduced with lentivirus carrying with shLacZ or TRIB3-specific shRNAs (#1 or #2) for 72 h. The mRNA expression of *TRIB3*, *ELF4*, *c-MYC*, and *CTNNB1* was determined by SYBR-Green-based qRT-PCR (**b**). ** *p* < 0.01; *** *p* < 0.001 when compared to shLacZ group. Specific amounts (25 μg) of total cellular proteins from TRIB3 knockdown EMC5 cells were used for examining the expression of β-catenin, c-Myc, BMI1, and NANOG by Western blot analysis (**c**). (**d**) The schematic diagram illustrates the role of TRIB3 in promoting EC progression through maintaining CSC activity by regulating the expression of β-catenin and other cancer stemness genes including BMI1, NANOG, and OCT4.

To further confirm the clinical role of TRIB3 in regulating cancer stemness, we collected five primary EC cells from Taiwanese patients; the characteristics of EC patients are listed in Table S1. We first observed the expression of TRIB3, β-catenin, and c-MYC in these primary EC cells (Figure 7a). EMC5, the established primary EC cells from patient No. 5 with clinical grade 3, was used as a model, and the knockdown of TRIB3 in these cells reduced the mRNA expression of *CTNNB1*, *MYC*, *ELF4*, (Figure 7b), and *MEF2A* (Figure S2d). The protein expressions of β-catenin, c-MYC, BMI1, and NANOG were also reduced in TRIB3-knockdown EMC5 cells (Figure 7c). These results revealed that the involvement of TRIB3 in regulating the β-catenin pathway and cancer stemness could also be observed clinically.

## 3. Discussion

It is known that the prognosis of EC is poor when recurrence and the recurrence rate of EC are associated with FIGO stage [5]. According to analysis of TCGA database, albeit not statistically significant, the expression of TRIB3 was found to be upregulated in EC patients as the stage increased (Figure 1b,c). We also found that the expression of TRIB3 was increased in EC tumorspheres and that the knockdown of TRIB3 caused a reduction in the CSC marker ALDH1A1, as well as cancer stemness genes (Figure 4c). The results from EC cell lines were also confirmed in primary EC cells (Figure 5b,c). These data suggest that TRIB3 may be a potential biomarker for EC. The main findings of this study were based on two type I EC cell lines of AN3CA and HEC1A. We also examined the role of TRIB3 in the proliferation and CSC activity of type II EC using a KLE cell line. After silencing of TRIB3 expression, the capabilities of colony formation (Figure S4a) and tumorsphere formation (Figure S4b) were greatly suppressed. In addition, the protein expressions of β-catenin, c-MYC, N-cadherin, and SNAI1 were reduced, while E-cadherin expression was increased, in TRIB3-knockdown KLE cells (Figure S4c). These data indicate that TRIB3 participates in the carcinogenesis of both type I and type II ECs.

Intrinsic and extrinsic stressors, including hypoxia, metabolism, reactive oxygen species, and inflammation, upregulate the CSC stress signaling pathway to enhance cancer cell survival and maintain cancer cell stemness [36]. TRIB3 can act as a stress sensor in response to a diverse range of stressors; thus, it participates in the pathogenesis of inflammatory and malignant diseases by interacting with specific intracellular signaling and functional proteins [9,37]. Hua et al. revealed that TRIB3 can interact with and stabilize the β-catenin–TCF4 complex and enhance colon cancer stem cell activity [15]. Activated β-catenin not only increases TRIB3 transcription but also supports TRIB3 stability, indicating a positive feedback loop between WNT/β-catenin signaling and TRIB3 expression in the induction and support of colon cancer stemness [15]. However, the regulation of β-catenin expression by TRIB3 in EC cells was not mediated by direct interaction (Figure 5f); rather, the regulation was mediated via both transcriptional (Figure 5c) and translational (Figure 5e) mechanisms. We also observed a significant positive correlation between *TRIB3* and *CTNNB1* expression at the mRNA level in ECs from TCGA database (Figure 5b, left panel). Furthermore, TRIB3 was also positively correlated with downstream targets of β-catenin, including *c-MYC* and *CCND1*, at the mRNA level (Figure 5b middle panel and right panel). Previous studies reported that several transcription factors, such as TCF4 [38], NK2 homeobox 5 (NKX2-5) [39], thyroid hormone receptor beta (TRβ) [40], and zinc finger protein 191 (ZNF191) [41], are responsible for the transcription of β-catenin. However, the transcriptional regulation mechanism of the β-catenin gene in EC is not fully understood. Here, we identified ELF4 and MEF2A as transcriptional activators that regulate β-catenin gene expression in EC. The expression of *ELF4* and *MEF2A* was obviously inhibited in TRIB3-knockdown EC cell lines (Figure 6d and Figure S2c) and primary EC cells (Figure 7b). MEF2A has been identified as a transcriptional activator that participates in several cellular processes, including neural differentiation, muscle development, cell growth control, and apoptosis [42,43]. ELF4 belongs to the ETS family of transcription factors, which also regulate cellular differentiation, proliferation, and transformation and promote tumorigenesis [44]. We analyzed data from TCGA database with the GEPIA webtool, and we found a significantly positive correlation between *MEF2A* and *CTNNB1* (*R* = 0.67, *p* = 0, Figure S2b) and between *ELF4* and *CTNNB1* (*R* = 0.56, *p* = 1.3 × 10^−15^, Figure 6c). A positive correlation between *MEF2A* and *TRIB3* (*R* = 0.15, *p* = 0.048, Figure S2a) and between *ELF4* and *TRIB3* (*R* = 0.21, *p* = 0.0052, Figure 6b) was also observed. In addition, we verified the binding of endogenous TRIB3 on three putative ELF4 binding regions within the *CTNNB1* promoter in EC cells (Figure 6f). Taken together, we substantiated that TRIB3 not only participated in the regulation of ELF4 but also cooperated with ELF4 in the transcriptional regulation of β-catenin. We hypothesize that ELF4 may function as a novel β-catenin transcription activator in EC cells through the help of TRIB3, a scaffolding protein massively located in the cell nucleus.

Our previous study revealed that TRIB3 can interact with DDX5/BNIP/BCLAF1 in radioresistant MDA-MB-231 cells; thus, it is involved in the self-renewal and radioresistance of triple-negative breast cancer cells by regulating Notch1 activation [14]. In this study, we also found that the activation of Notch1 was significantly inhibited in TRIB3-silenced EC cells (Figure S3). Wang et al. reported that miR-34a functioned as a tumor suppressor microRNA (miRNA) in EC cells by targeting Notch1 [45]. The overexpression of miR-34a or knockdown of Notch1 in EC cells reduced EMT features [45]. In addition to Notch1, the activation of the WNT/β-catenin pathway induces EMT in cancers [46]. The inhibition of EMT characteristics by TRIB3 knockdown in EC cells could result from the inactivation of Notch1 or β-catenin but remains to be further studied in the future, and the TRIB3-related signalosome in EC is worthy of further investigation using cell lines and primary EC cells.

## 4. Materials and Methods

### 4.1. Endometrial Cancer Cell Lines

The type I EC cell lines, AN3CA and HEC1A, and a type II EC cell line, KLE, were obtained from American Type Culture Collection (ATCC) (Manassas, VA, USA) and cultured in standard conditions according to ATCC instructions. All cell lines were validated with short tandem repeat analysis by the Center for Genomic Medicine, National Cheng Kung University (Tainan, Taiwan).

### 4.2. Establishment of Primary EC Cell Lines from Patients’ Endometrial Cancer Tissue

The collection of EC specimens was approved by the Institutional Review Boards at Chung Medical University Hospital (Taichung, Taiwan) with an approval No. CS18180. Primary human EC cells were isolated from fresh EC surgical specimens of Taiwanese female patients by enzymatic dissociation with collagenase/hyaluronidase solution (STEMCELL Technologies Inc., Vancouver, BC, Canada) followed by tumorsphere cultivation. The formed secondary tumorspheres were dissociated into a single-cell suspension (named EMC) using HyQTase solution (Hyclone Laboratories Inc., South Logan, UT, USA) and were maintained in a 37 °C, 5% CO_2_ incubator in Dulbecco’s modified Eagle medium (DMEM, GIBCO, Thermo Fisher Scientific, Waltham, MA, USA) supplemented with 10% fetal bovine serum (FBS, Hyclone Laboratories Inc., South Logan, UT, USA).

### 4.3. TRIB3 Knockdown with Lentiviral shRNA

pCMVΔ8.91, pMD.G, and the gene-specific shRNA (TRIB3#1:TRCN0000307989; TRIB3#2:TRCN0000295920); LacZ: TRCN0000231722) plasmids were purchased from National RNAi Core Facility (Academia Sinica, Taipei, Taiwan). Lentivirus production and transduction into cells were performed as described by our previous report [47]. Lentiviruses were generated by co-transfecting HEK-293T cells with a plasmid DNA mixture of shRNA (2.5 μg), pCMVΔ8.91 (2.25 μg), and pMD.G (0.25 μg) using HyFect^TM^ DNA transfection reagent (Leadgene, Tainan, Taiwan). Viruses containing media were then harvested at 48 h post transfection, filtered through a 0.45 μm filter, and used for transduction at 30% confluence with 8 μg/mL polybrene (Sigma-Aldrich, St. Louis, MO, USA) for 24 h. The medium was replaced with fresh medium supplemented with 2 μg/mL puromycin (TOKU-E, Bellingham, WA, USA) to select the successfully transduced cells.

### 4.4. Tumorsphere Cultivation

Tumorphere formation was performed as previously reported [14] The initial seeding cell number for primary or secondary tumorpshere cultivation was 2000 cells/well or 1000 cells/well, respectively. Briefly, 2000 EC cells were seeded into ultralow adherent six-well plates (Greiner Bio-One GmbH, Kremsmünster, Austria) with a tumorsphere medium containing 0.4% bovine serum albumin (Sigma-Aldrich, St. Louis, MO, USA), 20 ng/mL epidermal growth factor (PeproTech Asia, Rehovot, Israel), 20 ng/mL basic fibroblast growth factor, 5 μg/mL insulin (Sigma-Aldrich), 0.5× B27 supplement (GIBCO), and 4 μg/mL heparin (Sigma-Aldrich) and incubated at 37 °C and 5% CO_2_ for 10 days to form primary tumorspheres. The number of primary tumorspheres was counted under a phase-contrast microscope (Motic Asia. Hong Kong, China) using the 40× magnification lens. After counting, the primary tumorspheres were collected by a 70 μm cell strainer (BD Biosciences, San Jose, CA, USA), dissociated into a single-cell suspension with HyQTase solution (Hyclone Laboratories Inc., South Logan, UT, USA), and used for secondary tumorsphere cultivation following the protocol for primary tumorspheres except for an initial seeding cell number of 1000 cells/well.

### 4.5. Determination of Cell Growth and Colony Formation Assay

The trypan blue exclusion assay was applied for the analysis of cell growth at 24 to 96 h after cell seeding. Briefly, cells were seeded into a 12-well plate at a density of 1 × 10^4^ cells/well and the cell number was counted every 24 h after staining with trypan-blue (Gibco, Thermo Fisher Scientific Inc., Shanghai, China). The long-term cell proliferation was determined using a clonogenic assay. Briefly, cells were seeded into 12-well plates at a density of 250 cells/well and cultured for 7 days. The cell colonies were visualized after fixation with 2% formaldehyde followed by staining with 1% crystal violet (Sigma-Aldrich) for 2 h at room temperature, and the colony numbers were counted.

### 4.6. Western Blot and Coimmunoprecipitation (Co-IP)

Cells were lysed using the RIPA buffer with a proteinase inhibitor and phosphatase inhibitor cocktail (cOmplete^TM^, Roche, Basel, Schweiz), and 25 μg of total cellular proteins were separated by SDS-PAGE and transferred onto a PVDF membrane (Pall Corporation, Washington, NY, USA) followed by blocking with 5% skimmed milk in Tris-buffered saline/0.1% Tween-20 (TBS-T) buffer. The expressions of specific proteins were detected by incubation of specific primary antibodies followed by peroxidase-conjugated secondary antibodies, visualized by chemiluminescence substrate (PerkinElmer Inc., Waltham, MA, USA), and captured by the Luminescence-Image Analyzer (FUSION SOLO, Vilber Lourmat Deutschland GmbH, Wielandstraße, Germany). The composition of IP buffer was 20 mM HEPES (pH7.9), 2 mM MgCl_2_, 0.2 mM EDTA, 0.1 mM KCl, 1mM dithiothreitol, 10% glycerol, and 0.1% NP-40). For Co-IP analysis, 0.5–1.0 mg of total cellular proteins were incubated with 2 μg of specific antibody or normal rabbit IgG in IP buffer at 4 °C for overnight. Protein G Mag sepharose beads were used to precipitate the immune complex at 4 °C for 2 h followed by washing with IP buffer four times. The precipitated immune complexes were eluted in 25 μL of 2× sample buffer at 95 °C followed by separating with 10% SDS-PAGE and Western blot analysis as described above. The composition of 2× sample buffer was 125 mM Tris (pH 6.8), 4% SDS, 10% 2-mercaptoethanol, and 20% glycerol. The antibodies used in this study are listed in Table S2. All original blots images shows in Figures S5–S13.

### 4.7. RNA Isolation and Real-Time PCR

The purification of RNA, the synthesis of complementary DNA (cDNA), and the real-time PCR analysis were performed according to our previous report [14]. Total RNA was extracted and purified using an RNA extraction kit (Zymo Research, Irvine, CA, USA), and cDNA was synthesized with a first-strand cDNA synthesis kit (Fermentas Inc., Waltham, MA, USA). cDNA samples (10 ng) were used for the determination of gene expression with specific primers by SYBR Green Master Mix (Bio-Rad Laboratories, Inc., Hercules, CA, USA) with a PCRmax^TM^-Eco 48 Real-Time PCR System and analyzed with the Eco 48 software (PCR max, Staffordshire, UK). Each target gene was normalized to glyceraldehyde 3-phosphate dehydrogenase (GAPDH) to derive the change in Ct value (ΔCt). The changes in genes among groups were calculated using the 2^−ΔΔCt^ method. The primer sets used in this study are listed in Table 1.

### 4.8. RNA Sequencing Analysis

HEC1A cells were seeded in a 6 cm plate and transduced with LacZ or TRIB3-specific shRNAs carrying lentiviruses for 18 h followed by selection with 2 μg/mL puromycin for 3 days. Total RNA was extracted and purified using an RNA isolation kit (Zymo Research, Irvine, CA, USA). RNA samples were quality-controlled and sequenced by Biotools biotech Co., LTD. The raw reads were filtered by Trimomatic and then expression was normalized through RLE/TMM/FPKM. Differently expressed genes (upregulated and downregulated genes) were identified using a stringent cutoff value of twofold difference with an adjusted *p*-value < 0.05.

### 4.9. Migration and Invasion

The migration and invasion abilities of cells were evaluated using 24-well transwell chambers with 8 μm pore size of a polycarbonate membrane (Corning, NY, USA) by seeding 1 × 10^5^ AN3CA cells into the upper chamber in 100 μL of serum-free medium and embedding in the lower chamber containing 500 μL of 10% FBS-containing medium for incubation 24 h. The chambers were fixed with 4% formaldehyde and stained with 0.1% crystal violet. Non-migration cells were cleared with cotton swabs and photographed using an inverted light microscope (Motic, Schertz, TX, USA). For the invasion assay, the upper chamber was firstly coated with 0.5 mg/mL Matrigel (BD Biosciences, Franklin Lakes, NJ, USA) at 37 °C for 6 h.

### 4.10. Isolation of Cytosolic and Nuclear Fractions

Cells were pretreated with proteasome inhibitor, MG132, for 18 h and nuclear extract was isolated for the immunoprecipitation assay using the Nuclear Protein Extraction-Isolation Kit (FIVEphoton Biochemicals, San Diego, CA, USA) according to the manufacturer’s instructions with slight modification. The nucleus was lysed by NETN buffer and then sonicated for 30 s (on for 5 s, plus for 10 s). Nuclear extracts (200 µg) were used for the Co-IP experiment as described above.

### 4.11. Chromatin Immunoprecipitation (ChIP) qPCR

AN3CA and HEC1A cells were fixed for 10 min at room temperature in culture medium with formaldehyde (1% final concentration). Formaldehyde was quenched with glycine at a final concentration of 125 mM. Cells were washed twice with PBS, lysed in NETN buffer (150 mM NaCl, 1 mM EDTA, 20 mM Tris pH8.0, 0.5% NP-40) with a proteinase inhibitor and phosphatase inhibitor cocktail, and sonicated to shear chromatin to an average length of about 1 kb; DNA concentration was quantified as a function of A_260_ absorbance with NanoDrop. Chromatin DNA (75 μg) was used for immunoprecipitation with 3 μg of TRIB3 antibodies or normal rabbit IgG in dilution buffer (167 mM NaCl, 16.7 mM Tris (pH8.1), 1.2 mM EDTA, 1.1% Triton X-100, 0.01% SDS) at 4 °C overnight. Protein G Mag Sepharose beads were used to precipitate the antibody–chromatin complex at 4 °C for 2 h. Immunoprecipitated complexes were washed with low-salt buffer (150 mM NaCl, 20 mM Tris (pH 8.1), 2 mM EDTA, 1% Triton X-100, 0.1% SDS), high-salt buffer (500 mM NaCl, 20 mM Tris (pH 8.1), 2 mM EDTA, 1% Triton X-100, 0.1% SDS), LiCl buffer (250 mM LiCl, 10 mM Tris (pH 8.1), 1 mM EDTA, 1% sodium deoxycholate, 1% NP-40), and TE buffer (10 mM Tris, 1 mM EDTA), and eluted with 200 μL elution buffer for 15 min at room temperature. The reverse crosslink was performed by incubation of the samples overnight at 65 °C. After reverse crosslinking, samples were treated with RNase A in a final concentration of 0.5 μg/mL at 37 °C for 30 min, and then were treated with proteinase K in a final concentration of 50 μg/mL at 45 °C for 2 h. DNA was purified using a QIAquick PCR purification kit (QIAGEN, Hilden, Germany) according to the manufacturer’s instructions. Primer sequences used for qPCR detection are listed in Table S4.

### 4.12. NOD/SCID Xenograft Mouse Model

Single cells of primary tumorspheres derived from AN3CA cells were obtained after dissociation by HyQTase. A total of 5 × 10^5^ cells were suspended in 2.5 mg/mL Matrigel (BD Biosciences) and were subcutaneously injected into the interscapular area of NOD/SCID mice for tumor formation. The tumor formation was monitored twice per week, and tumor volumes were calculated with a formula of *D* × *d^2^/2* (*D*, length; *d*, width of tumor) [48]. The mice were sacrificed at Day 38 after cell injection, and the xenograft tumors were taken out for weighing and immunohistochemistry analysis.

### 4.13. Immunohistochemistry

Paraffin sections (5 μm) were deparaffinized and incubated with the primary antibodies (1:100 dilution) using a standard avidin–biotin–peroxidase complex method. The slides were incubated with 3,3′-diaminobenzidine (DAKO, Carpinteria, CA, USA) to detect the antibody binding. The slides were then counterstained with hematoxylin, mounted with Permount (Merck, Darmstadt, Germany), and scanned/analyzed with a TissueFAXS PLUS system (TissueGnostics, Vienna, Austria).

### 4.14. Statistical Analysis

The values of qRT-PCR, cell growth rate, colony formation, and tumorspheres were expressed as the mean ± SD (standard deviation). Significant differences between experiment groups or the tumor weight of xenografted tumors were determined using Student’s unpaired *t*-test at a 95% confidence level (GraphPad Prism version 5.0; GraphPad Software, La Jolla, CA, USA) or one-way ANOVA followed by Tukey–Kramer’s post hoc test (more than two groups).

### 4.15. Data Availability

The data that support the findings of this study are available from the following websites: The Human Protein Atlas (https://www.proteinatlas.org/) (accessed date: 13 December 2017) [29], SurvExpress (http://bioinformatica.mty.itesm.mx:8080/Biomatec/SurvivaX.jsp) (accessed date: 13 December 2017) [30], and TISIDB (http://cis.hku.hk/TISIDB) (accessed date: 23 November 2020) [31]; they are also available from the corresponding author on reasonable request.

## 5. Conclusions

Here, we discovered that the TRIB3 expression level is positively associated with a shorter overall survival of EC patients. The knockdown of TRIB3 in EC cells causes a reduction in cell proliferation, migration, invasion, CSC activity, and in vivo tumor formation through the inhibition of cancer stemness genes, such as BMI1, OCT4, Nanog, β-catenin, and c-MYC. The modulation of β-catenin expression by TRIB3 could result from translational regulation through the direct binding to GSK3β or from transcriptional regulation through the direct binding of ELF4 within the *CTNNB1* promoter to lead to the transcription of the *CTNNB1* gene (Figure 7d). These data clearly demonstrate that TRIB3 plays an oncogenic role in EC and positively regulates the self-renewal and tumorigenicity of EC-CSCs. Targeting TRIB3 is considered as a potential therapeutic strategy in future EC therapy.

## Figures and Tables

**Table 1:** Primers sequences used in this study.

Gene Name	Primer Sequence (5′ to 3′)
*TIRB3*	F: ACCGTATCCCTGAGCCTGA R: CTTGTCCCACAGGGAATCAT
*c-Myc*	F: AATGAAAAGGCCCCCAAGGTAGTTATCC R: GTCGTTTCCGCAACAAGTCCTCTTC
*CTNNB1*	F: GAAACGGCTTTCAGTTGAGC R: CTGGCCATATCCACCAGAGT
*MEF2A*	F: CAAGGGCATGATGCCTCCACTA R: GCTGAGTACACAAGTCCTTGCG
*ELF4*	F: AATTGGGACCGTCGCTAGACGA R: GTGGATGTTGCTGGGCACTGAA
*GAPDH*	F: CAATGACCCCTTCATTGACC R: TGGACTCCACGACGTACTCA

## Supplementary Materials

The following are available online at https://www.mdpi.com/2072-6694/12/12/3785/s1, Figure S1: The treatment of β-catenin inhibitors suppresses the cell growth of EC cell lines, Figure S2: TRIB3 regulates MEF2A expression in EC cells, Figure S3: TRIB3 knockdown reduces Notch1 activation, Figure S4: TRIB3 also participates in the cell proliferation and CSC activity of type II EC cells, Figure S5: The original blot images of Figure 2c, Figure S6: The original blot images of Figure 3c,d, Figure S7: The original blot images of Figure 5d, 5e, and 5f, Figure S8: The original blot images of Figure 6a,c, Figure S9: The original blot images of Figure 6e, Figure S10: The original blot image of Figure 6e, Figure S11: The original blot images of Figure 7a,c, Figure S12: The original blot images of Figure S2, Figure S13: The original blot images of Figure S3, Table S1: Characteristics of endometrial cancer patients, Table S2: Antibodies used in this study, Table S3: Primer sequences used in chromatin immunoprecipitation assay.
