# Supplementary material for: Tribbles Pseudokinase 3 Contributes to Cancer Stemness of Endometrial Cancer Cells by Regulating β-Catenin Expression
Source: Cancers (Basel). 2020 Dec 15;12(12):3785. doi: 10.3390/cancers12123785 (PMC7765506; doi:10.3390/cancers12123785)
Supplement: Supplementary file 1 [file cancers-12-03785-s001.pdf]

Supplementary Materials: Tribbles Pseudokinase 3 Contributes to Cancer Stemness of Endometrial Cancer Cells by Regulating β-Catenin Expression

Wen-Ling Wang, Guan-Ci Hong, Peng-Ju Chien, Yu-Hao Huang, Hsueh-Te Lee, Po-Hui Wang, Yueh-Chun Lee and Wen-Wei Chang

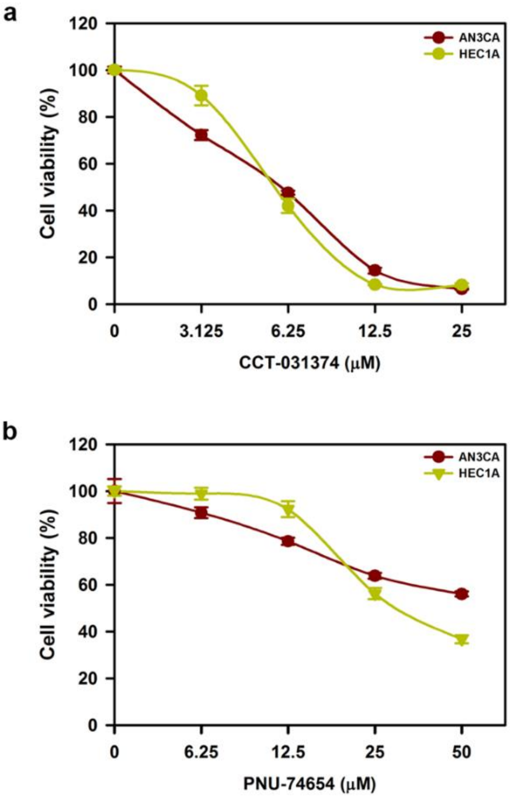

Figure S1. β-catenin pathway is involved in cell growth in EC cell lines. (**a**,**b**) AN3CA and HEC1A cells were treated with indicated concentration of β-catenin inhibitors, CCT-031374 (**a**) or PNU-74654 (**b**), for 72 hours and cell viability was determined by MTT reagent. Data represented relative cell growth to vehicle control (mean ± S.D.).

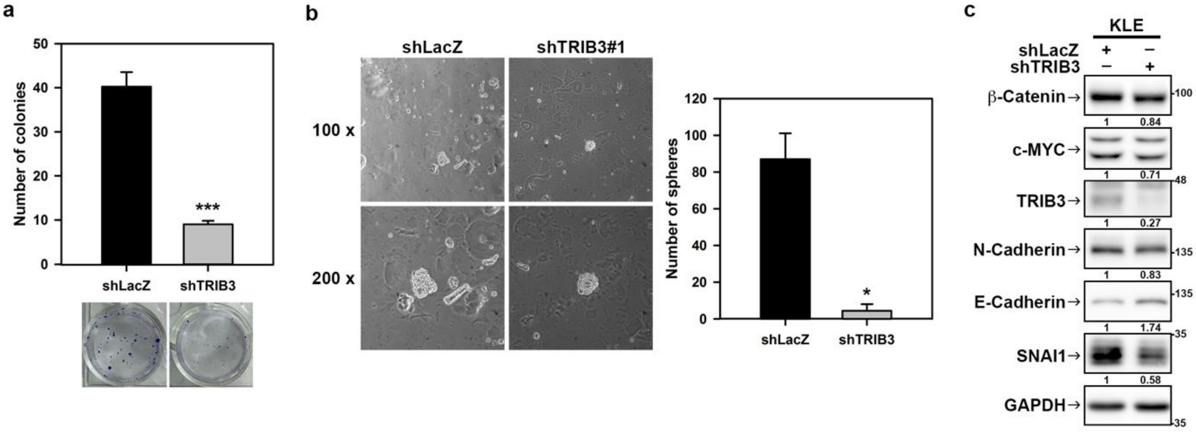

Figure S2. TRIB3 also participates to the cell proliferation and CSC activity of type II EC cells. KLE cells were transduced with lentiviruses carrying sh-LacZ or sh-TIRB3#1 shRNAs, respectively, and selected with 2 μg/mL puromycin for three days. (**a**) The survived cells were seeded into 12 well-plate

as 500 cells/well and incubated at 37 °C for 2 weeks. The formed colonies were visualized and counted after crystal violet stain. Data are presented as mean ± S.D. ***$p < 0.001$ when compared to sh-LacZ transduced cells. (**b**) The survival cells were seeded into ultra-low attachment 6-well-plate as 5000 cells/well and then performed tumorsphere cultivation for 4 weeks. The formed tumospheres were counted under an inverted microscopy. Data are presented as mean ± S.D. *$p < 0.05$ when compared to sh-LacZ transduced cells. (**c**) 25 µg of total cellular proteins from shRNA transduced KLE cells used for determining the expression of indicated proteins by western blot analysis. Original blot images are provided in Figure S12.

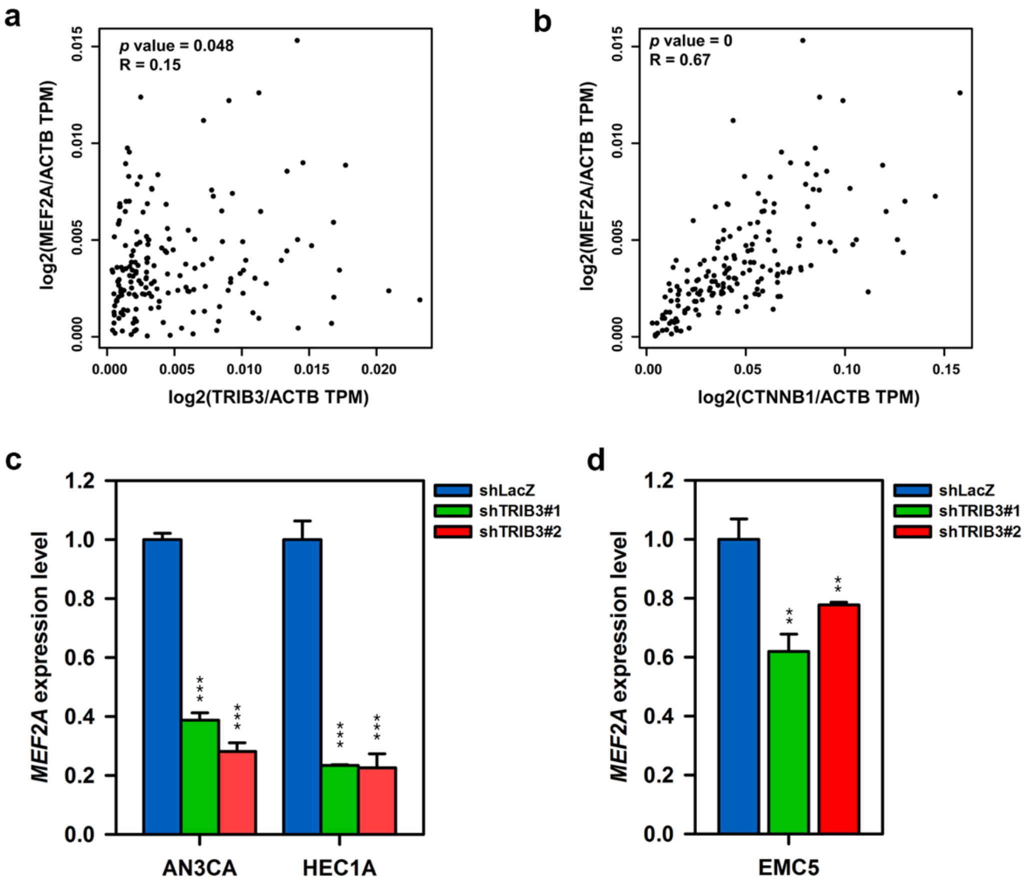

Figure S3. TRIB3 regulated MEF2A expression in EC cells**.** (**a**,**b**) Pair-wise correlations between TRIB3 with MEF2A (**a**) and between MEF2A and CTNNB1 (**b**) in UCEC patients were analyzed and plotted by GEPIA website. (**c**) The mRNA expression of *MEF2A* in TRIB3-knockdown AN3CA and HEC1A cells was analyzed by SYBR-Green based qRT-PCR. ***, $p < 0.001$ when compared to shLacZ group. (**d**) EMC5 cells were established from Pt5 and transduced with lentivirus carrying with shLacZ or TRIB3 specific shRNAs (#1 or #2) for 72 hours. The mRNA expression of *MEF2A* was determined by SYBR-Green based qRT-PCR. **, $p < 0.01$; ***, $p < 0.001$ when compared to shLacZ.

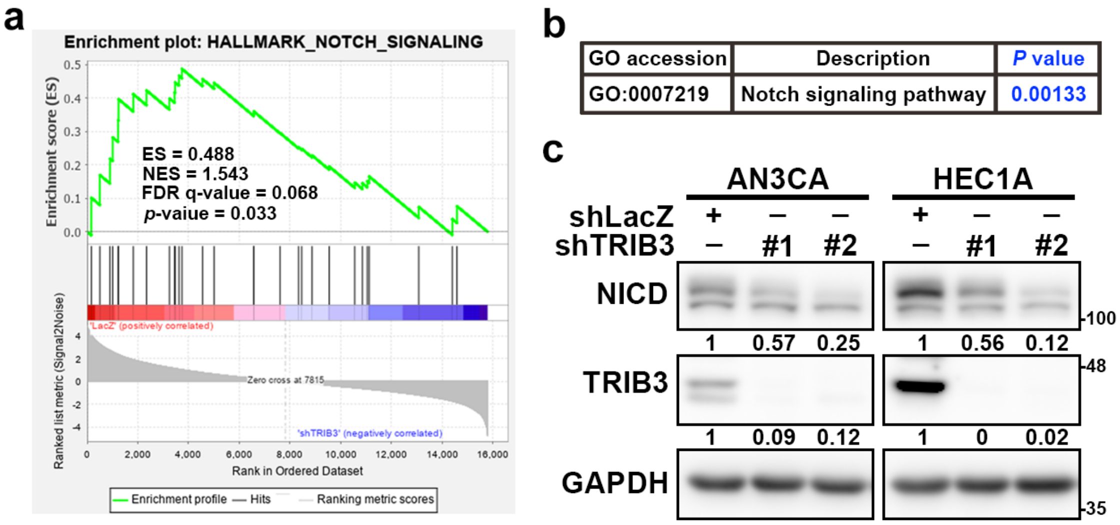

Figure S4. TRIB3 knockdown reduced activation of NOTCH1. (**a**) Gene set enrichment analysis of NOTCH signaling pathway related genes over shLacZ control (red) and TRIB3-knockdown (blue) form RNAseq data. (**b**) The genes downregulated in HEC1A cells with TRIB3 knockdown significantly enriched in NOTCH signaling pathway. (**c**)AN3CA or HEC1A cells were transduced with sh-LacZ or TRIB3 specific shRNAs (#1 and #2) carrying lentiviruses and selected with puromycin 2 µg/mL for 3 days. 25 µg of total lysate were used for the determination of indicated proteins by western blot analysis. Original blot images of Figure S4c are provided in Figure S13.

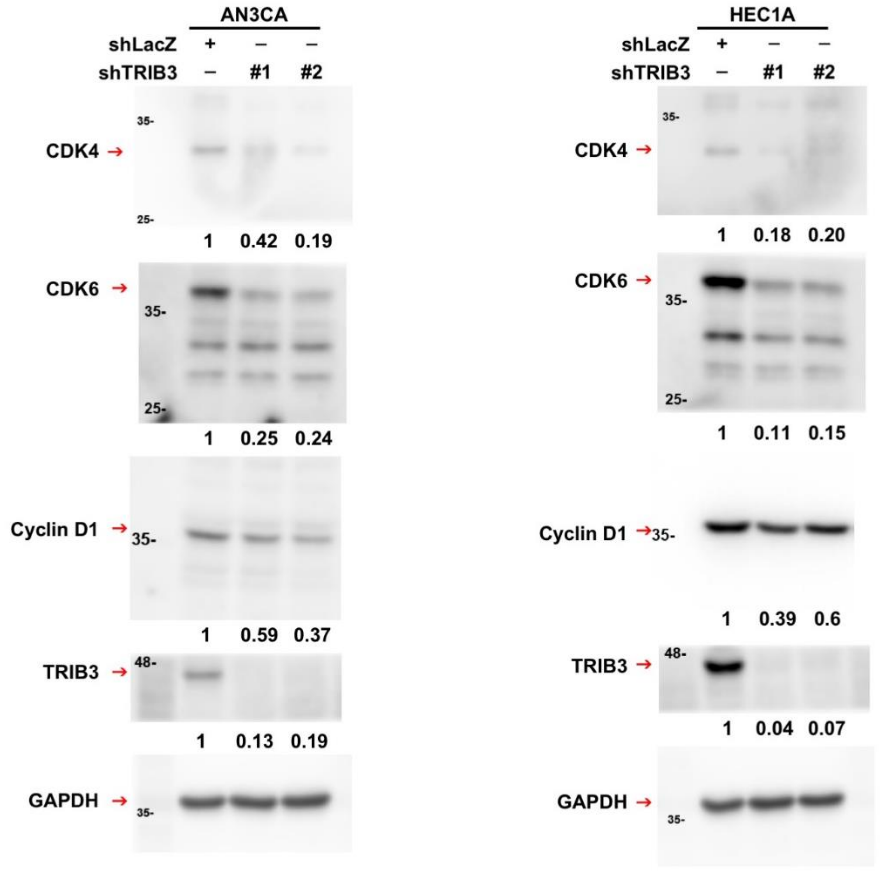

Figure S5. The original blot images of Figure 2c.

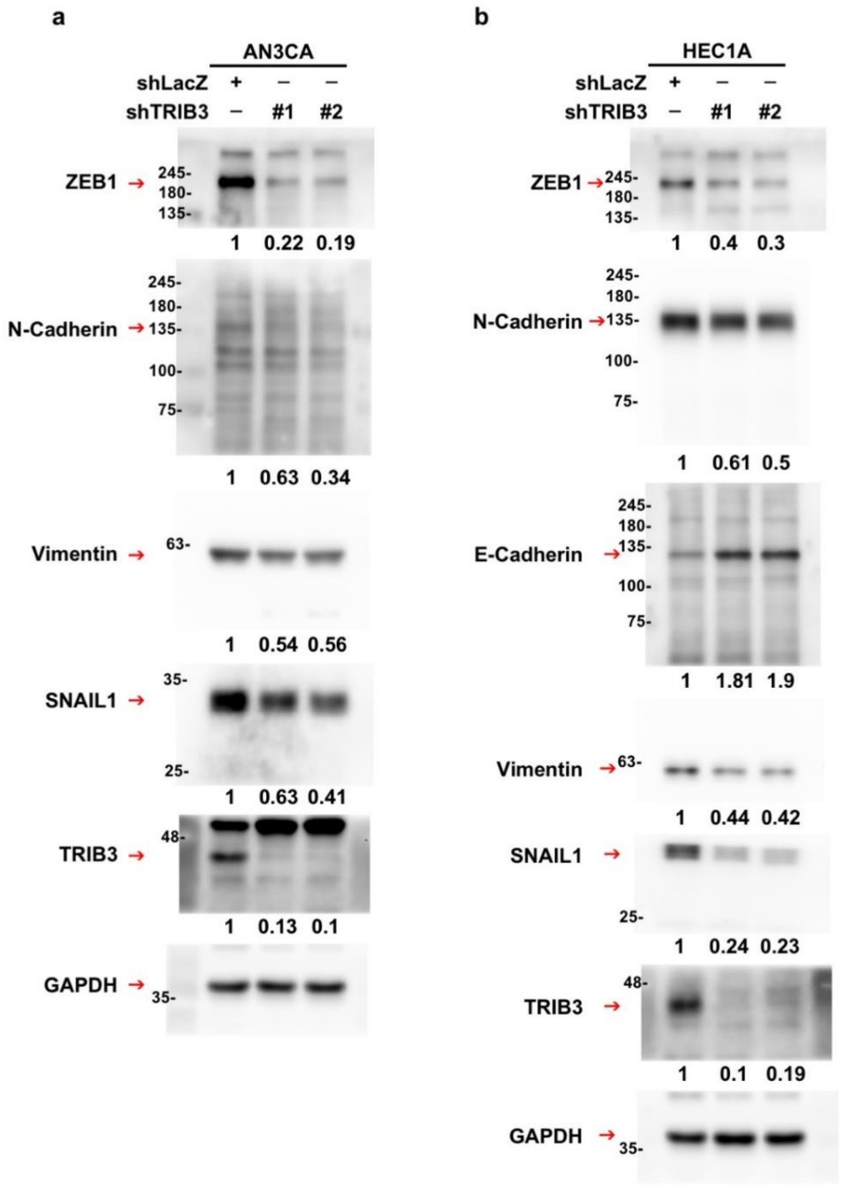

Figure S6. The original blot images of Figure 3c and 3d.

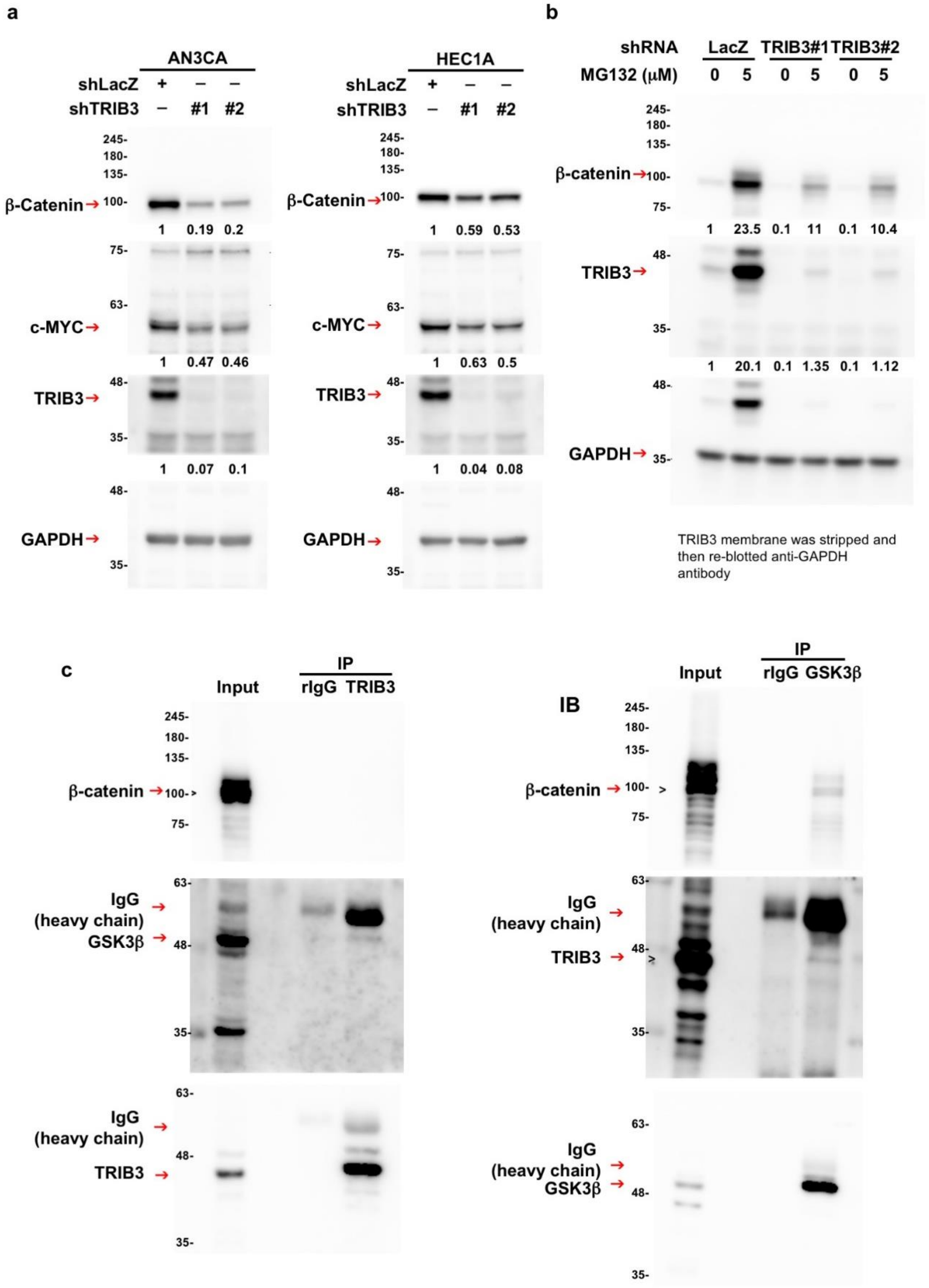

Figure S7. The original blot images of Figure 5d, 5e, and 5f.

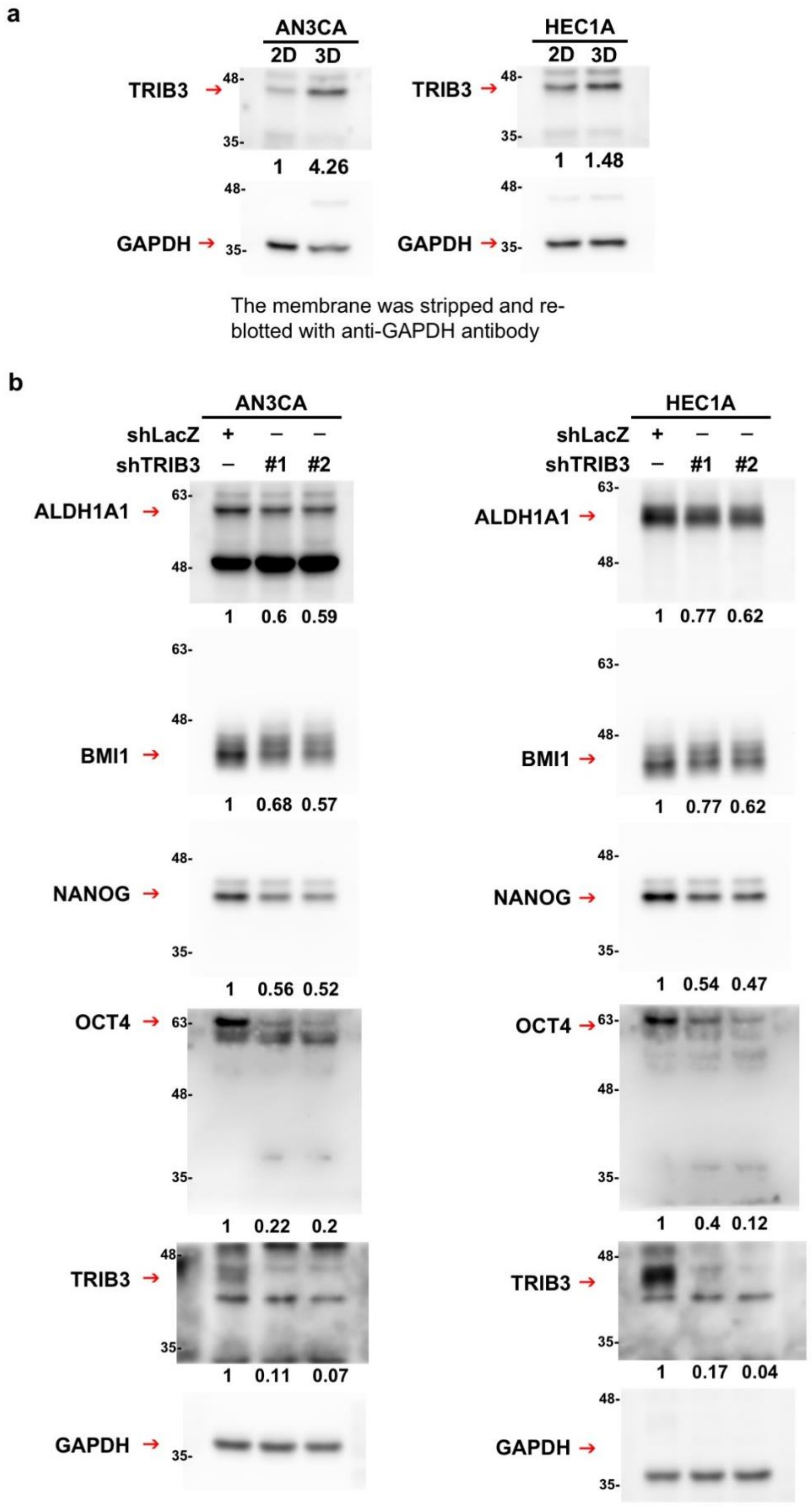

Figure S8. The original blot images of Figure 6a and 6c.

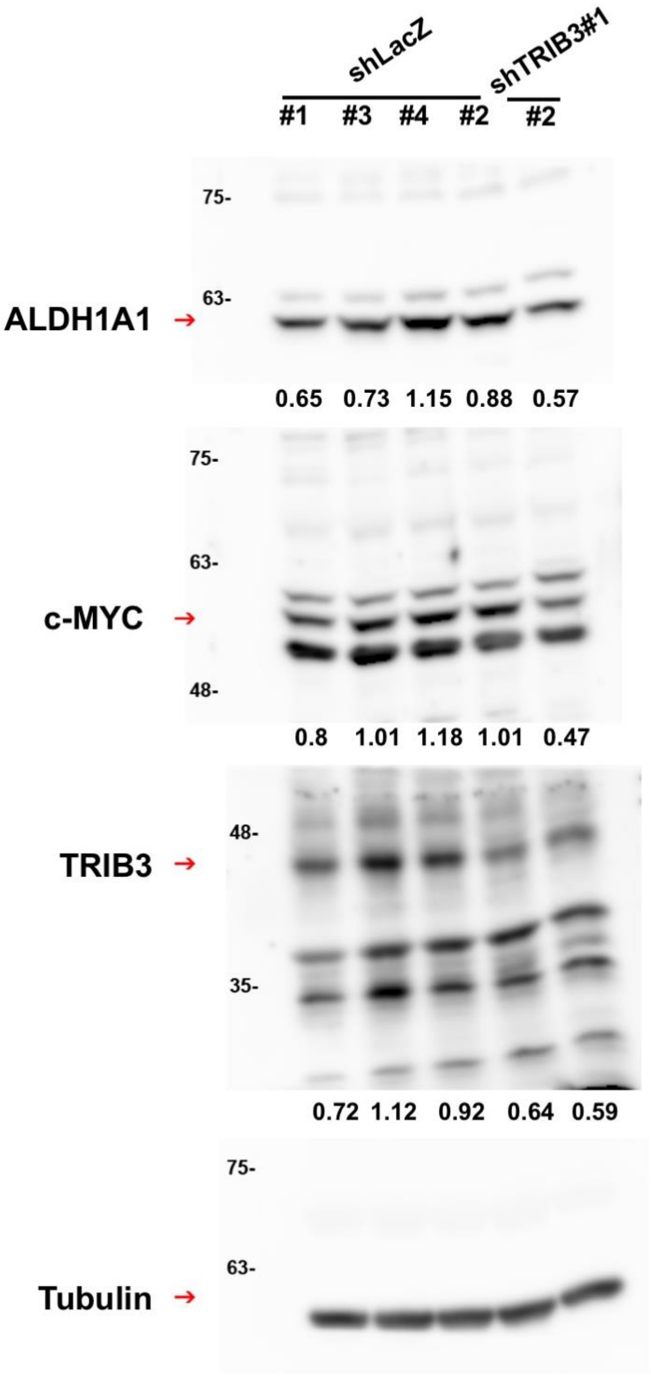

Figure S9. The original blot images of Figure 6e.

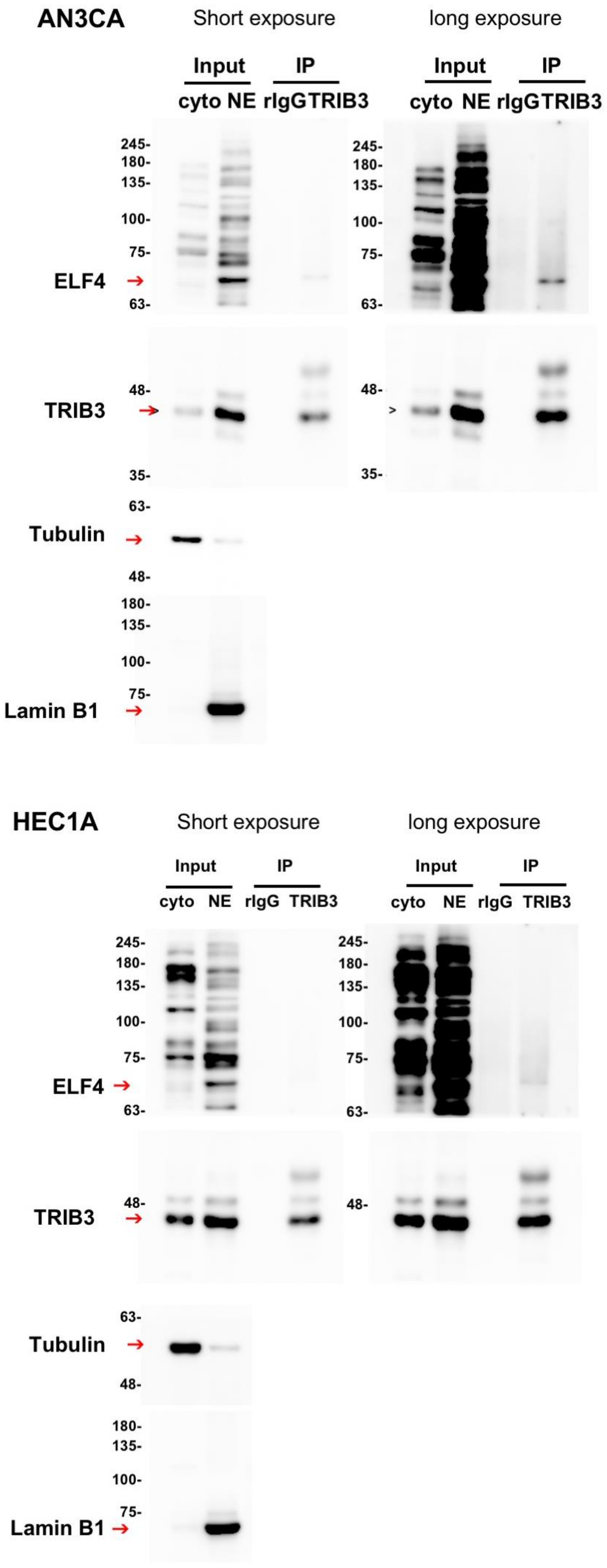

Figure S10. The original blot image of Figure 6e.

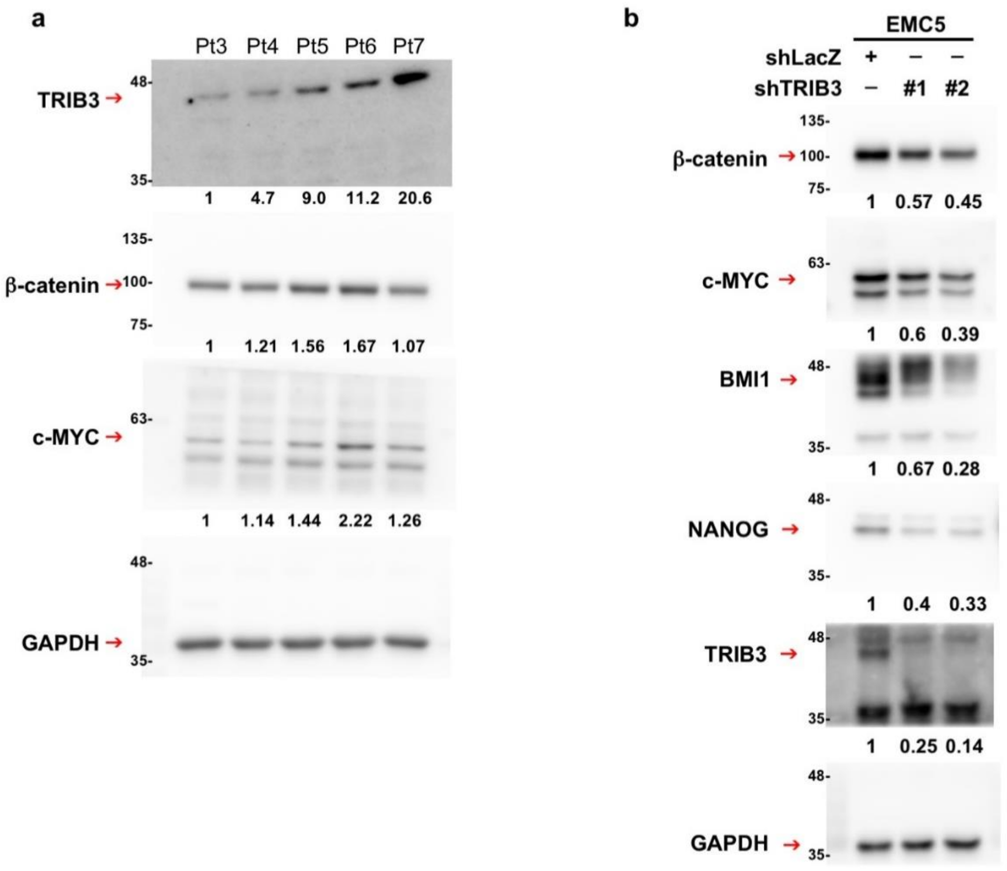

Figure S11. The original blot images of Figure 7a and 7c.

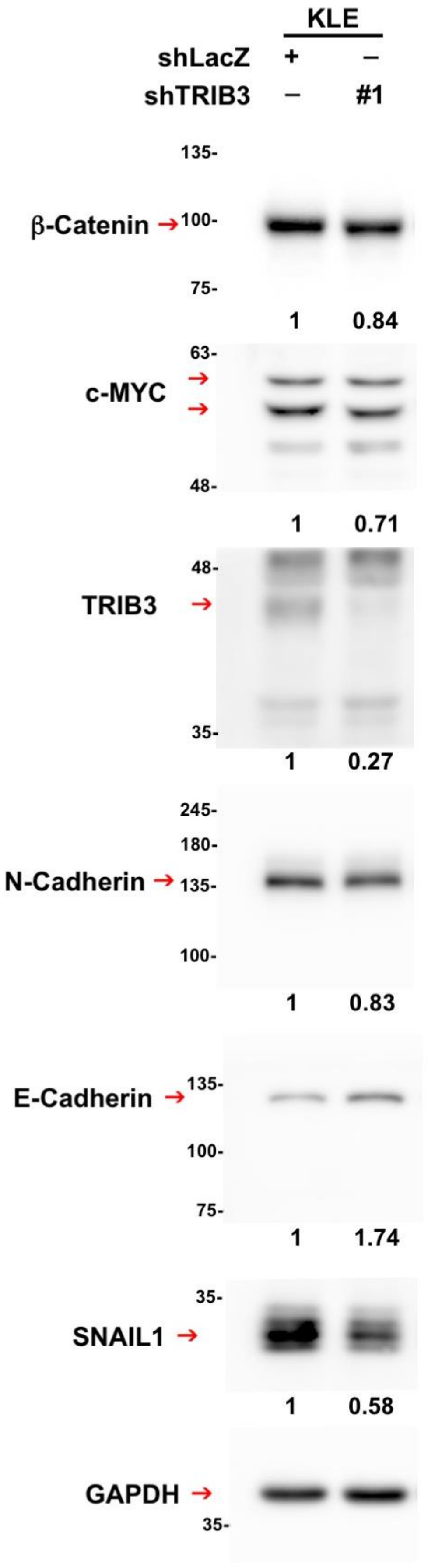

Figure S12. The original blot images of Figure S2.

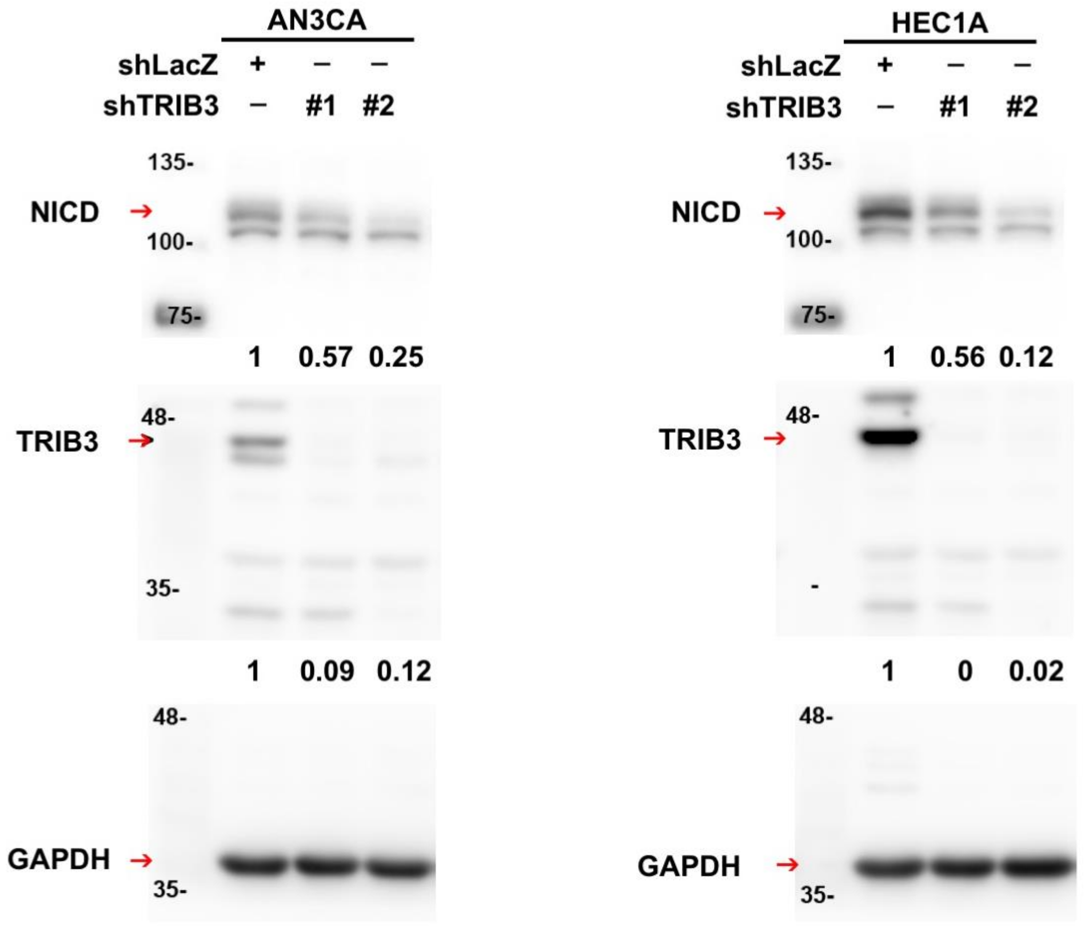

Figure S13. The original blot images of Figure S3.

Table S1. Characteristics of endometrial cancer patients.

Patient	Sex/Age	Histologic Grade	TNM	Primary Localization	ER*	PR*
Pt3	F/52	grade 2	T1aN1a	uterine fundus	90%	30%
Pt4	F/49	grade 1	T1a	uterine body	90%	90%
Pt5	F/60	grade 3	T3N1M0	Fragile filled in uterus	50%	40%
Pt6	F/60	grade 2	T1aN0M0	uterine fundus	80%	50%
Pt7	F/37	grade 2	T1aN0M0	uterine fundus, body and lower uterine segment	80%	90%

*ER: Estrogen Receptor; PR: Progesterone Receptor.

Table S2. Antibodies used in this study.

Product	Source	No. of Catalogue
Primary antibody:		
Western blot:		
anti-ALDH1A1	GeneTex International Corporation	GTX123973
anti-BMI1	Cell Signaling Technology, Inc.	6964s
anti-CDK4	Santa Cruz Biotechnology, Inc	sc-23896
anti-CDK6	Santa Cruz Biotechnology, Inc	sc-7961
anti-Cyclin A2	GeneTex International Corporation	GTX103042
anti-Cyclin B1	Proteintech group Inc.	55004-1-AP
anti-CyclinD1	IReal biotechnology	IR117-294
anti-c-MYC	GeneTex International Corporation	GTX109636
anti-⊚-Catenin	BD Biosciences	BD610154
anti-NOTCH1	Abcam plc.	ab2752b

anti-NANOG	Santa Cruz Biotechnology, Inc	sc-293121
anti-E-Cadherin	Santa Cruz Biotechnology, Inc	sc-21791
anti-N-Cadherin	Santa Cruz Biotechnology, Inc	sc-59987
anti-GSK3◎	Proteintech Group Inc.	15113-1-AP
anti-SNAIL1	Cell Signaling Technology, Inc.	3879s
anti-OCT3/4	Santa Cruz Biotechnology, Inc	sc-365509
anti-SOX2 (D9B8N)	Novus Biologiocals	NB110-37235
anti-TRIB3	Proteintech Group Inc.	13300-1-AP
anti-Vimentin	Santa Cruz Biotechnology, Inc	sc-66001
anti-ZEB1	GeneTex International Corporation	GTX105278
anti-Tubulin	Proteintech group Inc.	66031-1-Ig
anti-GAPDH	GeneTex International Corporation	GTX100118
Anti-ELF4	Santa Cruz Biotechnology, Inc	sc-515363
Immunohistochemistry:		
Anti-Ki-67	Abcam plc.	ab16667
anti-TRIB3	Proteintech Group Inc.	13300-1-AP
Chromatin Immunoprecipitation:		
anti-TRIB3	Proteintech Group Inc.	13300-1-AP
anti-GSK3◎	Proteintech Group Inc.	15113-1-AP
Secondary antibodies:		
anti-rabbit IgG-HRP	Jackson ImmunoResearch Laboratories Inc.	111-035-003
anti-mouse IgG-HRP	Jackson ImmunoResearch Laboratories Inc.	115-035-003
anti-rabbit IgG-HRP	GeneTex International Corporation	GTX221666-01
anti-mouse IgG-HRP	GeneTex International Corporation	GTX221667-01

Table S3. Primers sequences used in Chromatin immunoprecipitation assay.

ELF4 Binding Motif	Primer Sequence (5′ to 3′)
ELF4 P1 (−1349)	F: TACGCTGGCCCTGAAACATG<br>R:CCTCTGTGCTTTTATCCCAGG
ELF4 P2 (−650)	F: CTTTGGGGGTGCTGTGAGA<br>R: TCGCTGGTCTGCGGGTT
ELF4 P3 (+40)	F: GCGCCATTTTAAGCCTCTCG<br>R: CTGAAGCTGCTCCTCAGACC

Publisher's Note: MDPI stays neutral with regard to jurisdictional claims in published maps and institutional affiliations.

© 2020 by the authors. Licensee MDPI, Basel, Switzerland. This article is an open access article distributed under the terms and conditions of the Creative Commons Attribution (CC BY) license (http://creativecommons.org/licenses/by/4.0/).
